# Genomic evidence for fisheries-induced evolution in Eastern Baltic cod

**DOI:** 10.1126/sciadv.adr9889

**Published:** 2025-06-25

**Authors:** Kwi Young Han, Reid S. Brennan, Christopher T. Monk, Sissel Jentoft, Cecilia Helmerson, Jan Dierking, Karin Hüssy, Érika Endo Kokubun, Janina Fuss, Ben Krause-Kyora, Tonny B. Thomsen, Benjamin D. Heredia, Thorsten B. H. Reusch

**Affiliations:** ^1^ GEOMAR Helmholtz Centre for Ocean Research Kiel, Kiel, Germany.; ^2^ Centre for Ecological and Evolutionary Synthesis (CEES), Department of Biosciences, University of Oslo, Oslo, Norway.; ^3^ National Institute of Aquatic Resources, Technical University of Denmark, Kgs. Lyngby, Denmark.; ^4^ Institute of Clinical Molecular Biology, Kiel University, Kiel 24105, Germany.; ^5^ Geological Survey of Denmark and Greenland, Copenhagen, Denmark.

## Abstract

Overfishing is one human-driven perturbation driving major evolutionary pressure on marine populations. Fishing is often highly selective for particular traits and elicits marked phenotypic changes, while the evolutionary basis of such trait change remains unresolved. Here, we used a unique time series of the overexploited Eastern Baltic cod (*Gadus morhua*) to investigate growth trends during 25 years of heavy fishing along with hypothesized genetic changes at the full genome level. A growth analysis demonstrated a 48% decrease in asymptotic body length from 1996 to 2019 while a genome-wide association analysis revealed outlier loci and gene candidates linked to growth performance. The contributing loci showed signals of directional selection with high autocovariance of allele frequency change and significant overlap with regions of high genetic differentiation. Our findings suggest a genomic basis of fisheries-driven growth impairment and underscore implications for conservation policy regarding the adaptive potential of marine populations.

## Teaser

Growth decline and genomic shifts in Eastern Baltic cod reveal evolutionary response under fishing pressure.

## INTRODUCTION

Human beings play a substantial ecological and evolutionary role as they manipulate and disrupt environments and organisms by habitat alteration, pollution, climate change, and harvesting (*1*). It has now been widely acknowledged that, along with demographic population declines and community compositional shifts, such perturbations also elicit rapid evolutionary responses in wild populations (*1*–*3*). Rapid evolutionary changes caused by anthropogenic pressures, e.g., overfishing, pose special challenges in detecting induced selection processes, as the changes usually span a relatively short time frame (corresponding to a few fish generations) insufficient for a conventional sweep-like pattern caused by the complete fixation of focal alleles. Under these circumstances, historical time-series samples provide a special opportunity for detecting evolution in action by enabling reconstruction of past allele frequency changes in genomic data (*4*). In the context of fisheries-induced evolution (FIE), one of the strongest human perturbations caused by size selective mortality onto a fish population, the evidence for genetic or genomic changes in exploited populations is limited. In terms of general genetic diversity, meta-analyses using 140 fish species have revealed that overfishing reduces the genetic diversity of fish stocks compared to less exploited control populations (*5*).

Even less is known on the genomic change underlying specific trait changes. The most compelling evidence for genome-level responses to overfishing comes from 40 years of annual time series data of Atlantic salmon. A clear decrease in age at maturity in Atlantic salmon was accompanied by directional change in the allele frequency of *vgll*3 gene (*6*), a large effect locus explaining 39% of the phenotypic variation (*7*), which was significantly correlated with fishing pressure for the target species as well as a food species in salmon aquacultures (*8*). However, as most traits under fishing-induced selection, such as growth rate or age and size at maturity, have a polygenic basis with a large number of small effect loci, challenges remain in both the identification of the contributing loci and the detection of subtle changes in frequency of the loci [see (*9*, *10*)].

**Fig. 1.**
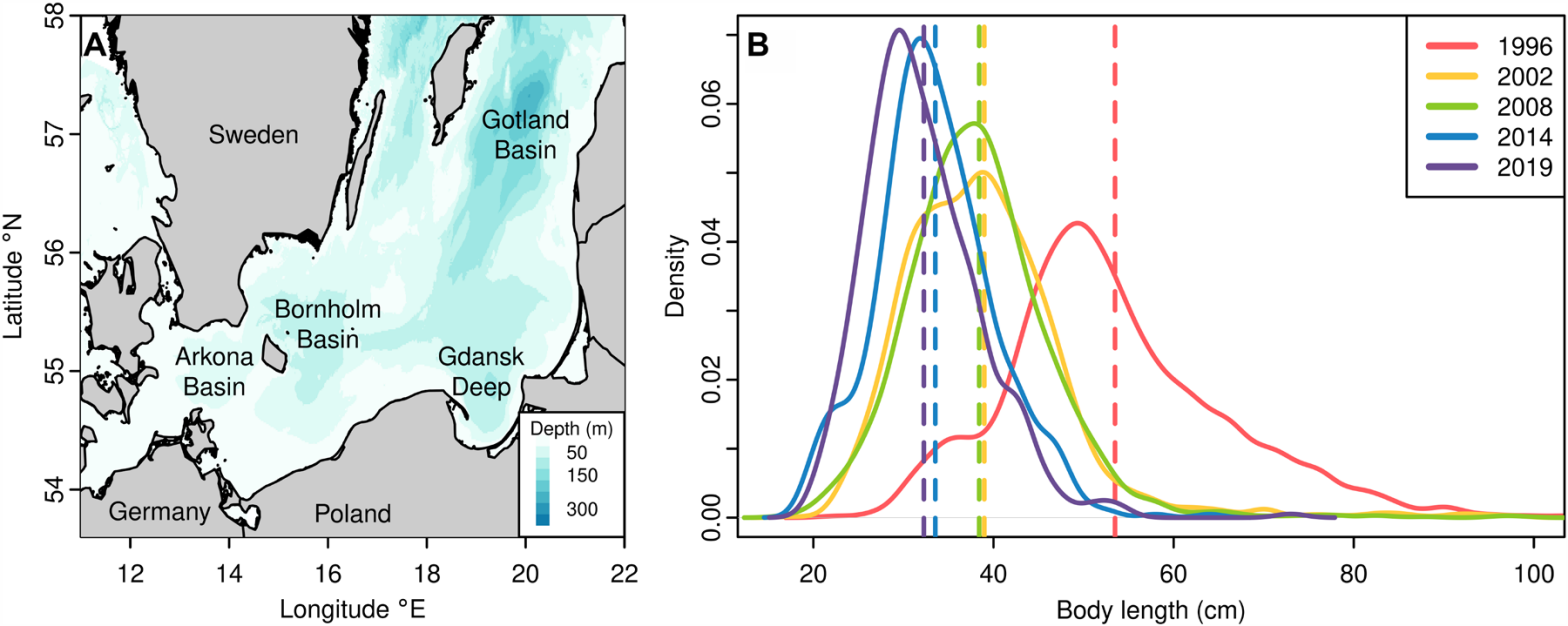
Sampling location in the Baltic Sea and length distribution of sample pools. (**A**) Map of the Baltic Sea showing the sampling sites in Bornholm Basin, the major spawning ground for EBC. Their historic spawning grounds in Gotland Basin and Gdansk Deep are not recognized as viable anymore. (**B**) Length distributions of all mature individual cods caught in Bornholm Basin in 1996, 2002, 2008, 2014, and 2019. Cod individuals were collected, and length and other phenotypic data were recorded on board of RV ALKOR in the Baltic Sea Integrative Long-Term Data Series. The vertical dashed lines present mean lengths for each catch year. Colors are according to the legend.

Eastern Baltic cod (EBC) is an Atlantic cod (*Gadus morhua*) population residing in the central Baltic Sea, with the last remaining spawning ground being the Bornholm Basin (Fig. 1A) (*11*). The population diverged from other Atlantic cod populations 7 to 8 thousand years ago when the Baltic Sea with its current salinity regime emerged after a series of postglacial tectonic shifts in combination with sea level changes (*12*, *13*). Now, it is biologically and genetically differentiated from all other ecotypes, e.g., western Baltic cod (WBC) and North Sea cod. It has adapted to the peculiar Baltic environment and experiences low salinities, high pCO_2_, prevalent hypoxia, and inconsistent and highly variable seasonal patterns of temperature, salinity, and oxygen contents (*14*–*16*). These fluctuating environmental conditions prevented the reliable conventional age reading via otolith rings, compromising the age-related data for stock assessments of EBC (*17*, *18*), which is why recent and historical data of age at maturity in this fish stock are unreliable. At present, EBC is genetically isolated from neighboring WBC due to the absence of genetic admixture (*19*, *20*), as indicated by the absence of hybrids in Arkona Basin, an area of spatial co-occurrence, in a recent 3-year study (*20*). This isolation is driven both by pre- and postzygotic isolation processes. For example, the eggs of WBC are not expected to survive in Bornholm Basin as they require much higher salinity for neutral buoyancy (*21*). Only during the historically unique population expansion of EBC in the 1980s, some limited hybridization between the EBC and WBC occurred (*22*). This makes the EBC population an ideal test case for coupling fisheries impact to genomic consequences by eliminating potential bias from migration and genetic admixture.

EBC plays a major role not only ecologically, being a key predator as the largest fish species in the Baltic Sea’s uniquely low biodiversity (*23*), but also economically. This population was the largest target for commercial fisheries in the Baltic with a peak annual catch of 400,000 tons in the mid-1980s (*11*). However, overfishing is well-documented with a fishing mortality typically two to three times higher than the maximum sustainable yield (*24*, *25*). Since the mid-1990s, multiple aspects of the EBC population have been deteriorating (*24*, *26*): The spawning stock biomass has declined sharply in recent years, together with declines in recruitment due to deteriorating oxygen conditions in deep water and the loss of two major spawning grounds (Fig. 1A) (*27*, *28*). Most markedly, L50 (length at 50% of population reaches maturity) declined from 40 to 20 cm and condition from 1.1 to 0.9 for 40- to 60-cm cod between 2019 and 2021 (*29*), along with a marked truncation of size distribution of mature adults (Fig. 1B). These biological changes have resulted in a ban on targeted fishing of EBC since 2019, but the condition of the population has not yet recovered to a healthy status.

Despite the prominent changes in the size composition of EBC, it is now unclear whether these shifts resulted from slow growth rate mediated by earlier maturation or deteriorating environmental conditions and/or higher mortality. The uncertainty in assessing the impact of these drivers is a consequence of unreliable age estimation for this stock. Unreliable age estimates have also hindered our ability to reconstruct probabilistic maturation reaction norms and estimate historic and current age at first maturation data for the population. In addition, whether these changes are driven by phenotypic plasticity or evolutionary change, i.e., FIE, has not yet been explored. In this study, we addressed this question by combining individual growth estimates based on novel age determination (i.e., chemical aging of otoliths) and whole-genome sequencing of individuals from multiple time points in the period of 1996–2019 (referred as “temporal population” hereafter). A sampling strategy to cover the full breadth of time and phenotype spectrum of the available sample pool was used. We first selected individuals randomly along the length distribution for each time point (a sample set called “random” hereafter). Then, to ensure a good representation of the extreme phenotypes, we also included individuals at both tails of the distribution (sample set “phenotype”). This unique dataset enabled us to explore the evolutionary basis of the phenotypic change observed within EBC over the last decades, examine the temporal genetic differentiation of the populations, and identify genomic regions of selection, which may have responded to pressure of overharvesting. Together, these results showcase the strength of combining temporal genomics of wild population with phenotypic data and provide insight into the genomic basis and consequences of FIE.

## RESULTS

### Temporal changes in growth rates

**Fig. 2.**
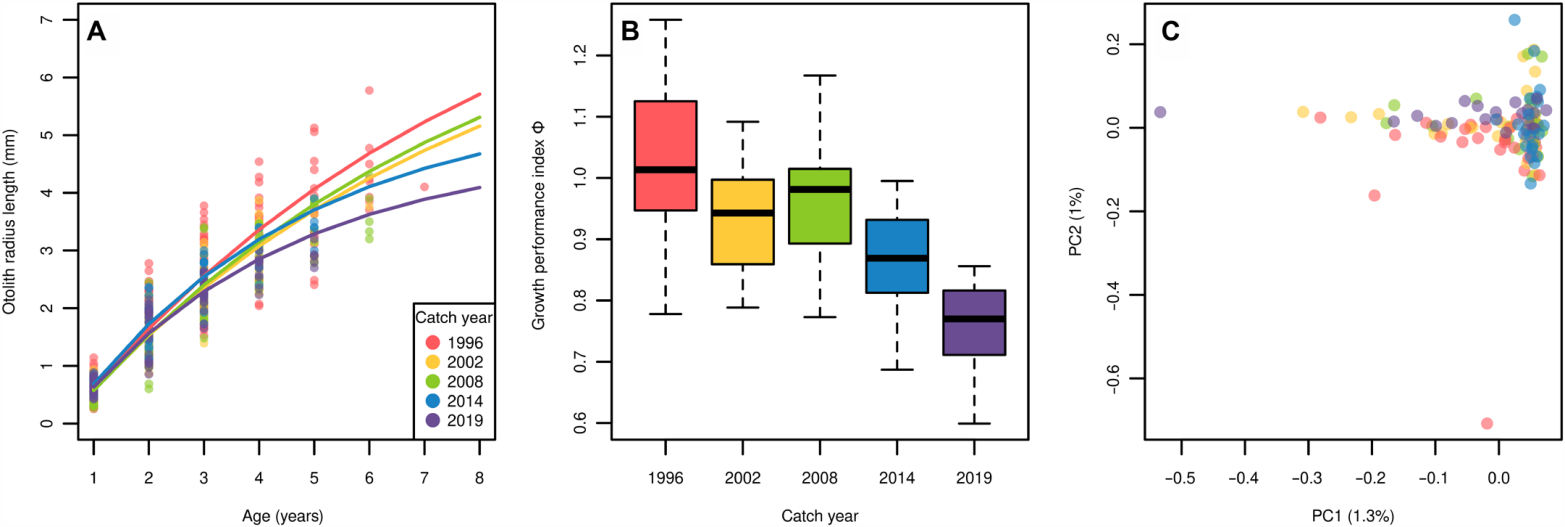
Population response over time. (**A**) Estimated von Bertalanffy growth curves for each catch year. The von Bertalanffy growth curves are based on otolith measurements and were plotted using estimated sets of parameters for each temporal population of the random and phenotype samples. The temporal group 1996 in this figure also includes the phenotype samples (catch year from 1996 to 1998) as they are treated as one temporal population in the model. Each point depicts observed otolith radius to chemical annuli at age colored on the basis of on the individuals’ catch year. (**B**) Boxplots of individual growth performance, Φ, calculated using estimated individual von Bertalanffy growth parameters (*L*_∞_ and *k*) over time. Color codes are based on individuals’ catch years as in the legend in (A). (**C**) Principal components analysis (PCA) of the 115 random samples. A set of SNPs was pruned on the basis of linkage disequilibrium and removed of sites within the inversions in LG2, LG7, and LG12. PC1 explains 1.3% and PC2 explains 1% of all variations in the genotypes. Each individual is coded in color according to the catch years as in the legend in (A).

To investigate phenotypic change under size-selective fishing pressure over the past 25 years (1996–2019), we focused on individual growth rates as the key heritable trait. We first aged archived otolith samples of 154 EBC individuals from Bornholm Basin using a novel method of age estimation based on seasonal patterns in the chronological records of otolith chemistry (*30*, *31*), a method that has been validated for EBC with an extensive tag-recapture program (*32*). The oldest fish was a 7-year-old caught in 1996, while individuals as old as 5 years old were sampled in more recent years (2014 and 2019) (table S1). Using the measured yearly otolith radii (for details, see Materials and Methods), von Bertalanffy growth parameters were estimated for each fish individually through time (table S2) (*33*). Fish in 1996 grew to reach a larger maximum size (*L_∞_*) and had a smaller Brody growth coefficient *k*, meaning they took longer to approach their terminal length than fish from recent years (Fig. 2A). The median of estimated individual length at infinity, *L_∞_*, decreased by 48% from 1996 to 2019, with a small inconsistency in 2008 (fig. S1A). Remarkably, this translates to a maximal fish length (*L_∞_*) decrease from 1150 mm in 1996 to 539 mm in 2019 using fish body length back-calculated from otolith radii. Accordingly, the growth coefficient *k* increased over the period, with the same anomaly in 2008, in both group parameters and individual parameters (fig. S1B). Growth performance index (Φ) for each fish of different years, which summarizes the growth (*34*), showed a consistent decrease across time (Fig. 2B). Additionally, the body length at age 1 was back-calculated for all fish from the corresponding otolith radii at age 1 and compared to examine any deviation in the juvenile growth of EBC in temporal trend (fig. S1C). Although mean distances to the first-year radii did not differ across time, the variance of the radii significantly reduced in more recent years (Bartlett’s test for variance, *P* = 0.03), indicating reduced phenotypic diversity in juvenile growth. The condition of individual fish [relative condition factor; (*35*)] showed statistically different population means only for 2002 (fig. S2A). When tested for correlation, individuals’ condition did not predict either of the growth parameters, *L_∞_* and *k*, or Φ [correlation coefficient (*r*) = −0.03, *r* = 0.09, and *r* = 0.09 respectively, all *P* > 0.05] (fig. S2, B to D). Overall, this supports that the population has shifted to reach smaller size when older via slower growth rates during the study period of heavy fishing pressure and that this was unrelated to the nutritional status of individuals. There also seem to be no issues with the larval and juvenile prey abundance, as growth rates were indistinguishable in year one among time intervals.

### Genome-wide temporal differentiation

To investigate any temporal differentiation of EBC that might potentially correspond to the phenotypic change, we subjected a set of single-nucleotide polymorphisms [SNPs; total of 5,847,389 SNPs of minor allele frequency (MAF) > 0.005] identified for 115 random samples to population summary statistics. First, a principal components analysis (PCA) using SNPs outside previously reported large chromosomal inversions revealed a panmictic population structure among time points (Fig. 2C). The variances explained by principal component 1 (PC1) and PC2 were relatively small (1.3 and 1%), while the loadings for each PC were well distributed along the whole genome. Second, we applied a temporal covariance analysis (*36*, *37*) to test genome-wide pattern of selection signature. The pairwise autocovariance of allele frequency changes in all time windows was indistinguishable from a simulated neutral scenario (figs. S3 and S4). The observed temporal autocovariance values from the samples were within the neutral distribution of drift-based changes (*P* > 0.05 for all paired autocovariances (fig. S5). Last, genome-wide nucleotide diversity (π) and absolute divergence between populations (*d_xy_*), calculated for 50-kb windows and varied only little among years (fig. S6). As expected, π and *d_xy_* varied along linkage groups (LGs) depending on differences in recombination rate along the chromosome, e.g., centromere regions featuring less recombination (*38*, *39*). Some divergence was observed at periphery of LG2 and in the central section of LG7, which were most likely caused by the varying frequency of the inverted regions (*40*). Overall pattern shows comparable genome-wide π (ranging from lowest value of 0.0071 for 1996 to highest value of 0.0077 for 2008) and consistent slight increase in *d_xy_* values as the sampling points are more distant in time, which likely indicates genetic drift over time.

### Genotype-phenotype association and identification of SNPs linked to growth

**Fig. 3.**
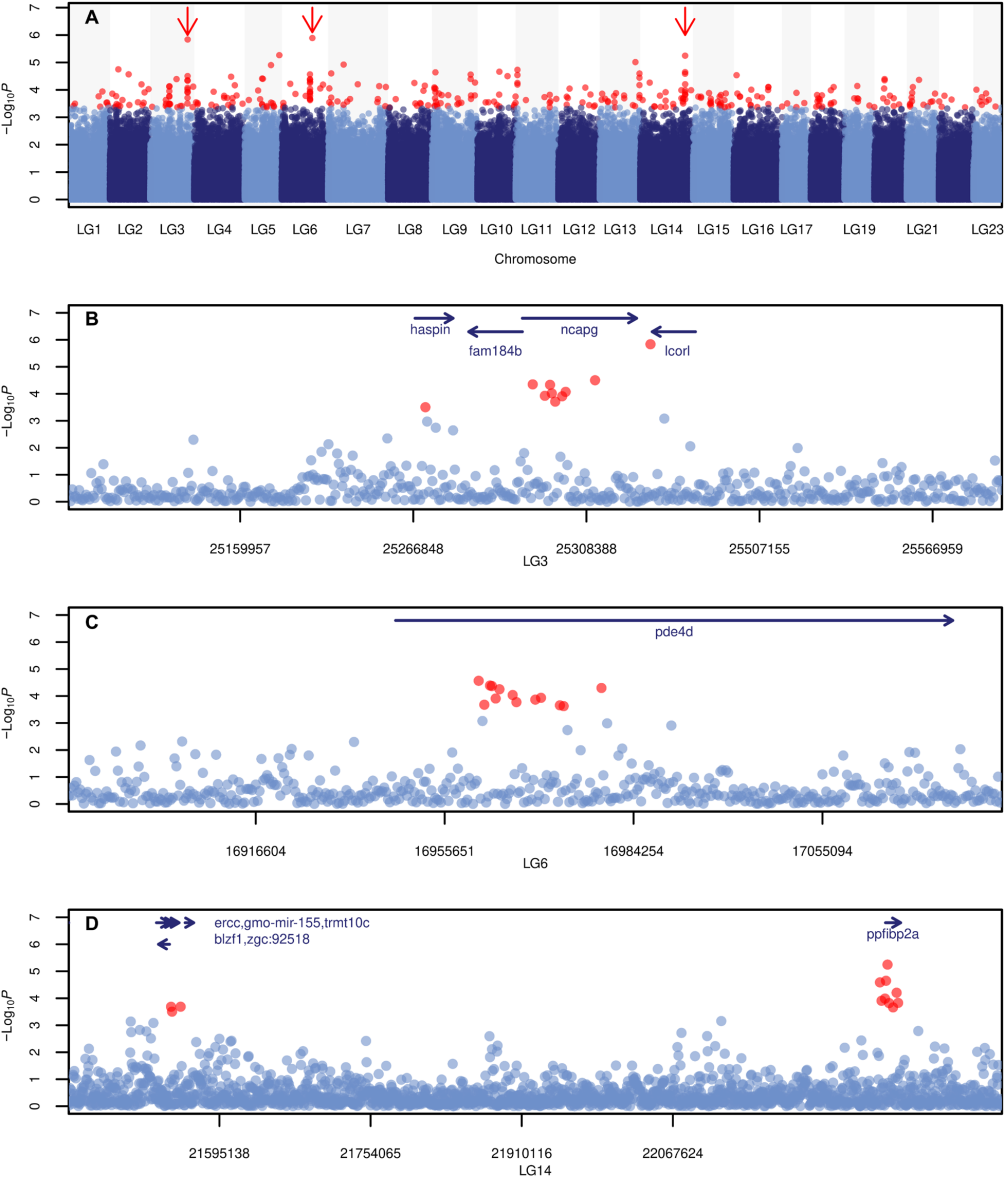
Manhattan plot of −log*P* values in GWA analysis. (**A**) Manhattan plot of −log*P* values in genome-wide association (GWA) analysis. A total of 152 samples were subjected to GWA using the sequenced genotypes, 679,584 SNPs (>0.05 MAF), and estimated growth performance index Φ as phenotype. Negative log-transformed Wald test *P* values for each SNP were plotted along the genome. Outlier status was assigned for 336 SNPs with lowest 0.05% *P* values (in red circles). The cutoff for outliers were selected on the basis of the visual examination of this Manhattan plot, so as to include distinctive peaks with clustering outliers (marked with red arrows) and, at the same time, exclude spurious outliers consisting of single SNPs only. In (**B**) LG3, (**C**) LG6, and (**D**) LG14, regions marked with red arrows were zoomed in, and genes residing at or near (5 kb up- and downstream) the outliers are annotated (Table 1).

Next, we sought to identify loci under directional selection by a genome-wide association (GWA) analysis using individual growth performance index (Φ) as a phenotype and 679,584 biallelic SNPs (MAF > 0.05) as genotypes. Three regions of the genome were clear outlier peaks with −log_10_*P* values of around 6 and most likely to be associated with growth performance (Fig. 3). Given the relatively low sample size, dictated by the availability of extractable DNA traces attached to historical otoliths, and the polygenic genetic basis of life history traits such as growth (*41*, *42*), we did not identify any loci with sufficiently large effect size upon multiple error correction. Instead, we consider the GWA analysis as an intermediate step to identify the polygenic signal of loci likely linked to the decrease growth rate. We then tested whether these loci were also under selection using further validation steps and a bespoke permutation test (*43*, *44*). To this end, we selected 336 SNPs representing the lowest 0.05% of the distribution of *P* values (see below).

First, regions with a peak of clustered outliers with flanking SNPs with low *P* values were examined in depth to seek biological relevance of the SNP sites. Genes located within 5 kb up- and downstream of the outliers were listed as candidate genes linked to growth variations (table S3). Among these candidate genes, the three most evident peaks of outliers in LG3, LG6, and LG14 contained genes that were most relevant to growth or maturity from functional annotation and previous research (Fig. 3, B to D): LG3 contains *ncapg*, which is differentially expressed in puberty in salmon (*45*), and *fam184b*, which is associated with body weight at first egg in chicken (*46*). LG6 included *pde4d* gene, which showed response in the transcriptome of fast growth line in a rainbow trout (*47*). Lastly, LG14 included *mettl21e*, which was linked to growth in pupfishes and intramuscular fat deposition in cattle (*48*, *49*).

Next, to understand whether the genomic regions explaining phenotypic variation were under selection through time, we calculated covariance values for the GWA outliers to observe directional change in their allele frequency. Specifically, lag-2 [i.e., cov(Δ1996–2008, Δ2002–2014) and cov(Δ2002–2014, Δ2008–2019)] and lag-3 [i.e., cov(Δ1996–2014, Δ2002–2019)] autocovariance (as illustrated in inlets of fig. S7) were calculated. Temporal covariances of allele frequency changes of 336 outlier SNPs exhibited remarkably high values of 0.00154 and 0.00187 for lag-2 and 0.00537 for lag-3 (fig. S7). Based on 1000 random permutations of covariance values of 336 SNPs sites, the observed covariances of GWA outliers markedly exceed the ranges of null distributions (*P* < 0.001, median value of 0.0002 and −0.0002 for lag-2 and 0.0002 for lag-3 autocovariance). This result strongly supports that the GWA outliers, highly correlated to the growth performance collectively, experienced selection and responded accordingly with a directional frequency change over time.

### Integration of the selection scan and GWA

**Fig. 4.**
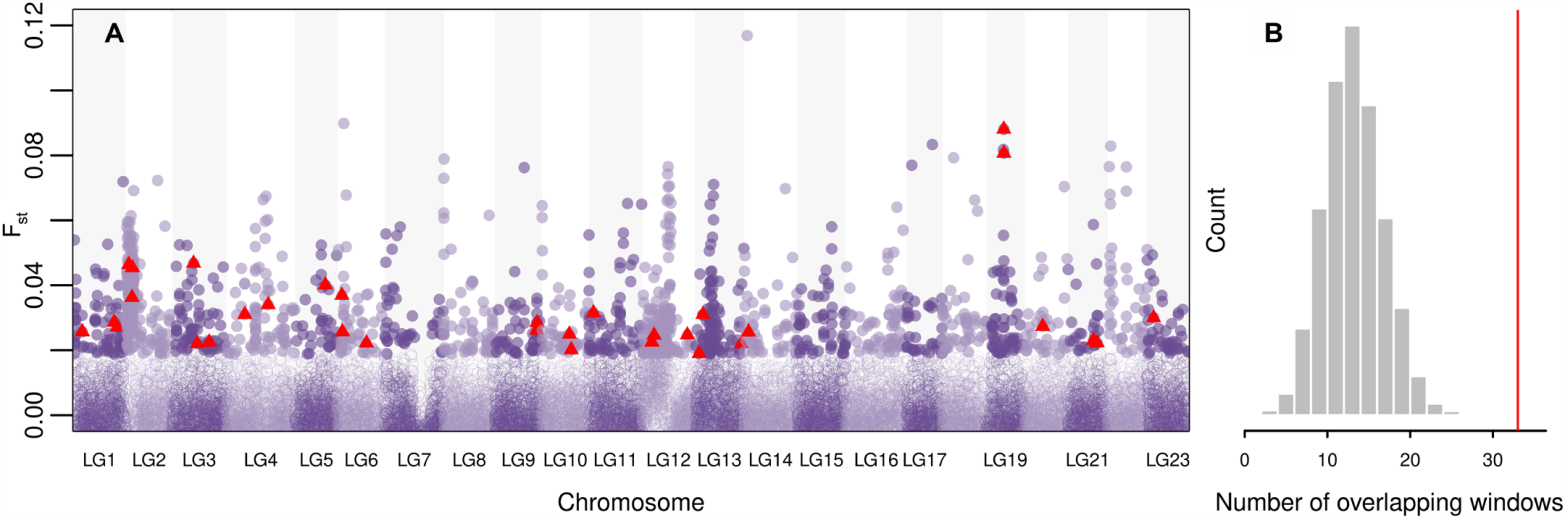
The integration of GWA results and F_st_ scan in 20-kb windows along the cod genome. (**A**) Pairwise F_st_ values of 1996 and 2019 in 20-kb nonoverlapping windows were calculated along the whole genome. Filled points indicate the highest top 5% of genome-wide F_st_ outlier windows. Among outlier windows, windows overlapping with GWA outliers are marked as red triangle. (**B**) The null distribution of the number of randomly overlapping windows of F_st_ and GWA outlier SNPs was drawn from a permutation test to assess the significance of the observed number of overlaps. The red line presents the observed number of overlapping windows in our dataset (*33*).

To further validate the 336 outlier SNP candidates, we combined the GWA results with a selection test in a complementary approach. A fixation index (F_st_) scan on 20-kb sliding windows across the genome was conducted comparing the temporal population of 1996 and 2019. Despite the lack of genome-wide signal of selection among temporal populations, we were able to identify regions of high differentiation (Fig. 4A). While the genome-wide F_st_ value was 0.001, which is a low value as expected for a single spatial population, some regions showed higher F_st_ values up to 0.1. When outlier windows of the 5% highest *P* values were compared with GWAS outliers, 33 windows overlapped. To test the statistical significance of this overlap, a null distribution was produced with a randomization test to which the observed values can be compared. Based on 5000 random permutations, wherein 336 SNPs were randomly chosen to compare with the outlier windows, the observed number exceeded the upper tail of the expected distribution (Fig. 4B). This signifies that loci associated with growth performance reside in the regions of highest F_st_ between 1996 and 2019. These two lines of evidence, the positive temporal covariance values of GWA outliers and their significant overlap with high F_st_ windows, strongly indicate the impact of directional selection on the genetic factors under growth variations in EBC.

The biological significance of these overlapping regions of GWA outliers and windows of high F_st_ was further explored through a gene ontology (GO) enrichment test (Table 1). Multiple pathways involved in ultradian rhythm, water homeostasis, protein metabolism, and meiotic cell cycle were enriched. Ultradian rhythm is important in diverse functions including growth, reproduction, and metabolism in fish (*50*, *51*). Diverse metabolic processes involving amino acids were also significantly enriched, which is critical for fish growth rates (*52*, *53*). Folic acid deficiency in diet has direct implications in fish growth as well as other functions such as immune responses. Pathways involved in mitotic cell cycle and development (e.g., regulation of mitotic cell cycle and embryonic, myotome development) together with multicellular organismal water homeostasis, which in common relate to a biological process called “oocyte maturation” form a large part of the list. In fish, oocyte maturation takes place before ovulation and is necessary for a successful fertilization (*54*), which may be indirectly linked to growth.

**Table 1.** A list of enriched GO terms using genes within F_st_ windows that overlap with GWA outlier SNPs. To identify loci that are highly correlated with growth performance and selected over time, the intersections of F_st_ outlier windows and GWA outlier SNPs were investigated. When a GWA outlier SNP resides within an F_st_ outlier window, this window was counted as an overlapping outlier window (marked as red in Fig. 4A). All genes residing within these overlapping outlier windows were subjected to gene ontology (GO) enrichment test to identify any biological functions that correlate to growth performance and, at the same time, differentiated the most over time. *P* values are adjusted using false discovery rate. Only biological processes were presented among GO categories for the analysis. CENP-A, centromere protein A.

GO term	GO name	*P* = value adjusted
GO:0007624	Ultradian rhythm	0.012
GO:0050891	Multicellular organismal water homeostasis	0.014
GO:0034080	CENP-A–containing chromatin assembly	0.014
GO:0006546	Glycine catabolic process	0.020
GO:0035825	Homologous recombination	0.021
GO:0007131	Reciprocal meiotic recombination	0.021
GO:0009396	Folic acid–containing compound biosynthetic process	0.022
GO:0009408	Response to heat	0.027
GO:0000122	Negative regulation of transcription by RNA polymerase II	0.033
GO:0009069	Serine family amino acid metabolic process	0.034
GO:0140013	Meiotic nuclear division	0.035
GO:1901606	Alpha-amino acid catabolic process	0.035
GO:0042558	Pteridine-containing compound metabolic process	0.035
GO:0061982	Meiosis I cell cycle process	0.035
GO:0051321	Meiotic cell cycle	0.046
GO:0001755	Neural crest cell migration	0.047

### Genomic regions of temporal selection

**Fig. 5.**
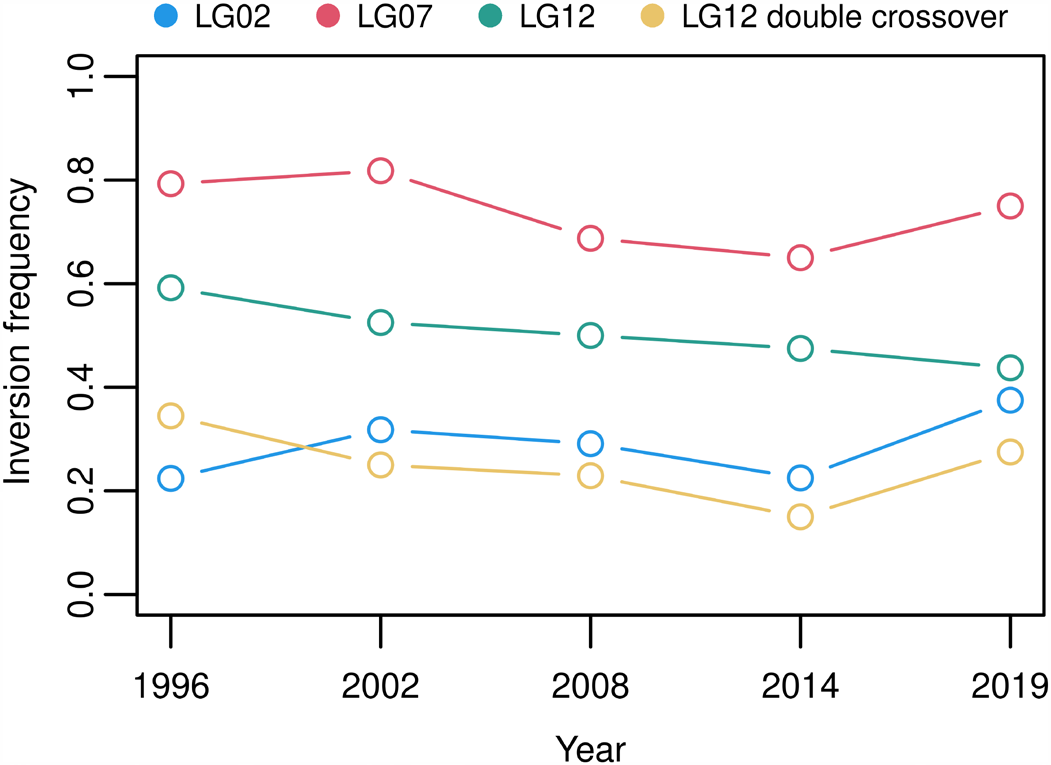
Frequency change of inversion haplotypes. The frequency of ancestral allele of each inverted region in LG2, LG7, and LG12 and the double crossover (DC) within LG12 are plotted over study period. The inversions in LG2 and LG7 display an inconsistent pattern. For the inversion in LG12, a monotonic decrease in frequency over time is observed that is statistically significant (Mann-Kendall test for monotonic trend, *P* = 0.03), whereas the frequency of DC within the region changes independently.

Along with selection signals observed in regions associated with growth phenotype, we were able to infer selection signatures in other parts of the genome. In the F_st_ selection scan, pronounced high F_st_ values in LG2 and LG12 as well as low values in LG7, where previously reported inversions reside (marked in pink Fig. 4), were observed along with highly conspicuous deviations of π values (fig. S6). Thus, we calculated the haplotype frequency for each inversion in the temporal populations. Only the inversion in LG12 was consistently decreasing in frequency over time (Mann-Kendall test for monotonic trend: *P* = 0.03) (Fig. 5). Within this inversion, another block of inverted region, so called “double crossover” (DC), was reported to be private to the EBC population (*12*). Thus, we identified the DC within the inversion in our sequence data (fig. S8) to examine the temporal trend of its frequency. Unlike the consistent decrease in the haplotype frequency of LG12, frequency of the DC within only decreases until 2014 and then picks up in 2019. So, it seems that, while the large inversion in LG12 shows a pattern of directional selection as a whole, the DC deviates from this pattern and shows a sign of drift or balancing selection on its own.

Additionally, the highest F_st_ values outside inverted regions were spread across the genome, some of which appear in peaks of clustered outliers. GO term enrichment analysis was conducted using 575 genes residing within the top 5% of F_st_ windows (table S4). Several enriched GO terms were highly related to the growth of a fish, including metabolism and the processing of macromolecules such as amino acids, fatty acids, and carbohydrates, which are central to energy utilization. Fatty acid oxidation was particularly notable due to its role in providing energy during feeding conditions (*55*), while cyclic adenosine 3′,5′-monophosphate biosynthesis is part of processing adenosine 5′-triphosphate, which is also critical in regulations of hormones involved in metabolism and reproductions (*56*). Also, regulation of target of Rapamycin (TOR) pathway, which is crucial in sensing growth hormone, nutrient, or oxygen condition (*57*, *58*), was found to be enriched.

## DISCUSSION

Whether or not FIE is “real” and not just phenotypic plasticity has sparked considerabe scientific debate that continues to this date [reviewed in (*59*, *60*)]. There has been a general lack of genomic evidence of FIE in the wild (*61*), either from limited detection power due to the polygenic nature of the traits of interest or insufficient data of adequate resolution, which has long frustrated scientists [see (*62*)]. Addressing this critical gap, this study highlights the genomic regions with associated gene functions that are correlated with growth impairment in an exploited marine fish population. Reassuringly, our candidate regions were also found to be under directional selection using genome scans and temporal covariance approaches, suggesting FIE in a wild population.

Obtaining sufficient biological replicates in studies involving historical and degenerated DNA is challenging. For quantitative traits, it is likely that the genetic underpinnings are polygenic, which means that, under realistic sample sizes for difficult to obtain historical samples, it is highly unlikely to have the statistical power to identify significant associations following multiple testing corrections (*63*). For example, quantitative traits in humans typically only explain a fraction trait variance despite leveraging samples sizes in the millions (*64*). Under these constraints, the combination of covariance and F_st_-based selection tests, preceded by a GWA to enrich for gene regions correlated with the target trait, here, growth rate, seems to be a promising way forward to study the temporal dynamics of polygenic traits under selection (*43*, *44*). This approach integrating association mapping and selection scans has been shown to work well to identify the genomic basis of polygenic traits underlying adaptation in multiple model and non-model systems (*65*–*67*).

### Overall patterns of temporal genomic change

We find that EBC is a closed, self-sustained gene pool without immigration of divergent genotypes, thus making it an ideal test case to study FIE (Fig. 2C and fig. S9). This is supported by the nonsignificant change of nucleotide diversity, a lack of clustering pattern in PCA, and neutral genome-wide covariance patterns. These results suggest that migration, gene flow, and other nonadaptive processes were negligible over the study period, in accordance with other recent marker-based studies (*20*).

The absence of evidence of genome-wide temporal changes does not equate to evidence of absence of selection (*43*, *65*). When looking more closely at specific genomic regions, we find nonrandom signals: the inverted region of LG12 and the candidate loci of GWA. Against the background of no overall change in genomic patterns (figs. S5 and S6), the directional change in the frequency of inversion in LG12 might suggest selection in parallel to the apparent decline of growth rates. In EBC, little evidence of adaptive or ecological roles of inversion haplotypes has been provided. Although no GO terms were found significantly enriched for genes located within the inverted region of LG12, the ancestral homozygous status of individuals, together with body size, had a correlation to lower survival rate in an Atlantic cod population in the North Sea (*40*). In addition, loci within this inverted region were highly correlated with temperature and oxygen level at the surface likely driving the differentiation of cod populations (*19*). The frequency of DC within the inverted region seems to be fluctuating independently of the large inversion. This region is densely packed with genes including three vitellogenin genes, which are crucial for creating buoyancy of eggs for the survival and successful spawning in EBC (*68*).

### Functional relevance of selected loci

Several enriched GO pathways for the overlapping regions of GWA and F_st_ outliers suggest that the selected gene functions could be causally linked to reduced growth rates in EBC (table S3). For example, in fish aquaculture, light manipulation to modulate the ultradian rhythm of individuals and, hence, their perception of season is a very common method to control growth and maturity, including Atlantic cod (*69*–*71*). Depending on the photoperiod applied, sexual maturation can be controlled, either postponed or advanced, which has marked implications for somatic growth rates (*71*, *72*). In addition, water homeostasis is important in egg hydration during the oocyte maturation process before ovulation to make more buoyant eggs, which is one of the major evolutionary acquisitions for pelagic teleost fish (*73*). While specific hypotheses connecting oocyte maturation with growth rates are now lacking, it is conceivable that oocyte maturation and the timing of spawning are critical for successful reproduction, as maturation process is highly affected by energy allocation (*74*). Last, the biological process of “response to heat” is highly linked to growth traits in fish. Rapid ocean warming in Baltic Sea by about 0.5°C decade^−1^ (*75*) critically affects the species throughout the life span from larva to adult stage (*76*, *77*) and is inversely proportional to oxygen availability. Thus, slow growth could also be accompanied by shifts in temperature response over time.

How do our results of multiple biologically interpretable gene candidates compare to earlier experimental results? A general lack of congruency among genetic change was observed even among replication lines within an experiment by Therkildsen *et al.* (*78*) who resequenced samples from the seminal study of Conover and Munch (*79*). In that experiment, Atlantic silversides were subjected to five generations of upward and downward size selection, respectively, compared to randomly selected controls. Their results of enriched GO terms under different harvesting regimes did not overlap to our study, which is perhaps not unexpected given that highly divergent genomic responses were observed across replicates under the same treatment in the study (*78*). In another experimental study, Uusi-Heikkilä *et al*. (*80*) identified genes in zebrafish that were selected by simulated fishing pressure; these genes were similarly nonoverlapping with the outliers in this and any other study [e.g., (*81*)]. This lack of consistency of selected FIE genes and pathways across these experiments underscores the polygenic nature of the trait and the heterogeneous genomic responses to the same selective pressure of size-selective fishing. The complexity of polygenic traits means that different sets of genes may respond to similar selective pressures, even under the same conditions or genetic background; that is, there is genetic redundancy (*82*). Genetic redundancy appears to be a major factor during rapid adaptation, limiting the extent of parallel genomic responses to selection despite similar phenotypic outcomes (*83*). Last, the *vgll3* and *six6* genes known for their high effect size for age at maturity in salmonid species (*7*, *84*), a distinct life history trait tightly linked to growth, were not found to be significant in any of the present analyses.

### Future research directions and implications in fisheries management

While we find significant evidence that loci associated with reduced growth rate are also under directional selection, definitively linking these loci to FIE is beyond the scope of the present study. While we suggest that future work should establish causative links between these candidate alleles and FIE, we here explore our results and their implications in the context of FIE and other perspectives of the population.

First, evolutionary responses occur in the context of dynamic interplay between fisheries and adverse environmental factors. Examples abound that overexploitation will cause evolutionary change (*81*), but these responses are context dependent. Depending on the life history traits under selection and their genomic architecture as well as the strength, length and types of selection pressure, natural selection by various environmental factors may reinforce or counteract the FIE in a convoluting manner. In the Baltic Sea, environmental factors, such as hypoxia and temperature have increased alongside other ecological variables, such as predator-prey interactions and interspecies competition; these changes have directly and indirectly influenced Baltic Cod in addition to fishing (*85*–*88*). These additional changes may have exerted additional evolutionary pressure resulting in phenotypic change. For example, sea surface temperature has risen around 1.5°C during the study period (*89*). However, this should only have caused a 6% decrease in body size according to gill-oxygen limitation theory (*90*), as compared the 48% reduction seen in Baltic Cod. Lack of oxygen, in its direct impact, triggers physiological stress response and alters feeding behaviors in cod (*91*–*93*). Otolith chemistry analysis of EBC has shown that growth and condition decrease as exposure to hypoxia increases over a fish’s lifetime, irrespective of sex and age (*87*, *94*). Oxygen availability in the bottom water in the Baltic Sea has been continuously deteriorating since the 1930s and the extent to which Bornholm Basin has been directly affected has been variable depending on inflow from North Sea and surrounding rivers (*16*, *95*). However, one would expect growth rates to be relatively equally affected across life stages in cod; here, we find that growth only decreased at older ages and was the same in the first year of life. Thus, the decrease in oxygen is unlikely to explain all of the growth reduction in this system. More work is needed using time-series data of adequate resolution to disentangle the impacts of fishing pressure and other environmental changes on growth.

Second, this study focuses on the critical period of a steep decline and lowest point in growth from 1996 to 2019 and provides a contemporary snapshot of the long-term population dynamics of EBC. However, growth rates have fluctuated over longer time scales, with an increase during the 1960s to 1980s, followed by a noticeable decline from the 1990s to the present (*96*). Thus, the direct causes and evolutionary responses shaping the growth trend warrant further investigation within a longer timeframe, ideally preceding and extending the study period. For examples, an inherent lag in evolutionary response, referred to as “Darwinian debt” (term coined by U. Dieckman in an interview by *Financial Times*), may be contributing to the delay of recovery by compromising growth potentials and population resilience (*59*, *97*). Darwinian debt reflects the long-term and accumulative evolutionary costs of rapid, human-induced selection, where adapted traits such as smaller adult size and earlier maturation will persist even after selective pressures have ceased. This delay in population-level trait reversal can hinder population recovery and highlights the pressing need for genetic monitoring and an assessment of evolutionary trade-offs over extended timescales in many overexploited fish stocks.

When we accept the genomic responses in EBC obtained here as an indication for an evolving population characterized by stunted growth, this study has clear implications for managing the stock. Successful management plans for EBC must incorporate evolutionary aspects into their framework, e.g., introducing *F*_evol_ (evolutionarily-sensitive threshold for fishing mortality) (*98*) and integrating evolutionary processes into economic assessments of management plans (*99*–*101*). At present, despite the current moratorium, the stock recovery falls short of expectations due to concurrent contribution of ecological and environmental factors to stock condition (*26*). Whether this lack of recovery is already one consequence of the Darwinian debt is an interesting hypothesis to explore in the future.

## MATERIALS AND METHODS

### Ethics statement

Tissue and otolith samples were obtained from individuals caught during routine fisheries surveys conducted through the research vessel ALKOR (IMO 8905880). No animals were captured to be maintained in an experimental facility. According to the German “Landesfischereiverordnung” (German Fisheries Act, §4 sec 4), a general permit is granted to research institutions such as GEOMAR Helmholtz Centre for Ocean Research Kiel to obtain fish samples for the purpose of scientific stock monitoring. These data directly feed into stock assessments through the ICES. Hence, no individual permit number will be issued.

### Sample collections

Otoliths and fin clips were collected in the Baltic Sea Integrative Long-Term Data Series of the Research Division Marine Evolutionary Ecology at GEOMAR, obtained by scientific trawl fishing on research cruises carried out annually since 1996. They were taken on board from cod caught in Bornholm Basin (Fig. 1A), of which their phenotype data (e.g., body length, weight, maturity stage, and sex) were recorded (table S1). Otoliths were stored in paper bags. Fin clips were stored in 97% ethanol at −20°C.

With this large pool of samples from this archive, sampling was done in two different ways to cover the available time period and the full range of phenotype in the sampling pool. (i) A set of samples, called random, was randomly sampled along the length distribution for five catch years; and (ii) another set of samples, called phenotype, contained smallest mature fish and largest immature fish from the catch year 1996–1998. As age information of the archived samples was not available, sample based neither on the cohort nor on length at first maturity was possible. The rationale was that, by sampling immature fish, which would be first mature in the following year if they had not been caught, and small, presumably young, mature fish, we attempted to cover as wide a range of phenotype variation as possible. As many of these otolith samples would contain degraded DNA or otherwise cross-contaminated—these samples are old, stored at room temperature and not originally intended for genomic analysis—we conducted a quality control of all DNA extracts for suitability in sequencing as well as cross contamination (see details below), where around 40% of the starting pool had passed. A total of 164 cod individuals were used in this study for age reading of otoliths and sequencing. Among these individuals, 10 phenotype samples (8 biggest immature and 2 smallest mature fish) were identified as WBC population from genetic analysis (fig. S9), and two samples resulted in low sequencing quality. Therefore, a total of 154 samples were used for growth analysis: random samples of 31 from 1996, 22 from 2002, 24 from 2008, 20 from 2014, and 20 from 2019 and phenotype samples of 19 smallest mature fish and 18 largest immature fish (table S1) and 152 samples for all the downstream analysis using sequence data.

### Age reading of otoliths

As the conventional otolith reading method has not been reliable for EBC, a recently developed method was used to acquire age information of the sequenced samples to model growth based on (*32*). This method makes use of seasonally recurring patterns in the chemical composition occurring in otolith from the core (corresponding to the time of hatch) to the otolith edge (corresponding to the time of capture). The concentration of certain elements reflects growth of the fish, and interpretation of chronological patterns of these elements, therefore, provides information of not only growth but also the age of the fish (*30*–*32*). The method was validated using tag-and-recapture samples of EBC population (*32*). A direct assessment of the accuracy of this approach is now not possible given the fact that known-age samples do not yet exist. However, an assessment of aging precision documented the superiority of chemical aging with an agreement of up to 80% between readers compared to an agreement of only 50% using traditional age interpretation. For the chemical analysis, otoliths were embedded in Epoxy resin (Struers) and sectioned transversely to expose a smooth surface from the core to the dorsal otolith edge. Trace element analysis was conducted by laser ablation inductively coupled plasma mass spectrometry (LA-ICP-MS) to measure magnesium (^25^Mg), phosphorus (^31^P), and calcium (^43^Ca), which exhibit seasonal variations in EBC (*31*, *32*). Because the element profiles (fig. S10) were interpreted from the core of an otolith to the edge, the measured element traces represent the chemical characteristics of an individual’s life span from the hatch to catch. Chemical minima were identified by first smoothing the element profiles with local polynomial regression function “loess,” followed by detection of minima in the smoothed profiles with the “peaks” package in R (R Development Core Team, 2022). The arguments were set based on the settings used in chemical aging of tag-and-recapture cod samples by Hüssy *et al.* (*32*). The numbers of minima in Mg and P, which suggest the fish’s exposure to the coldest temperature of a year (February and March), are counted, thereby obtaining the age of an individual [fig. S10 and figure 2 of (*32*)]. When the two values disagreed, the element profiles were visually examined. This generally occurred in samples caught at the beginning of the year, because this smoothing and minimum detection approach is sensitive to the number of measurements between the last minimum and the otolith edge. Thus, visual assessment was conducted for the samples caught in the first quarter of a year. To obtain annual growth rates, the distances between otolith core and each of the chemical minima along the dorsal otolith radius were extracted for each individual, together with total otolith radius, in addition to the age at catch. The exact details of preparation of otoliths, procedures concerning LA-ICP-MS, and the statistical analysis can be found in (*32*).

### Modeling individual growth rates

To acquire a heritable phenotype that may have been affected by fishing pressure, we modeled individual growth using the age information. Although it was recently confirmed that the growth of EBC has decreased over last decades (*96*), it is crucial to obtain the growth pattern of sequenced individuals to integrate genotype and phenotype.

To fully use the hierarchical nature of the data on otolith growth from core to chemical minima for consecutive ages of each fish, Bayesian hierarchical modeling was applied using R2jags v0.7.1 R package (*102*). The von Bertalanffy growth function (*33*) was fitted to distance from core to chemical annuli at age on otoliths from different catch years

$$L_{a}=L_{\infty }\left[1-e^{-k\left(t_{a}-t_{0}\right)}\right]$$

where *L*_a_ is distance from otolith core to each chemical annulus; *t*_a_ is the estimated age at the annulus; *L_∞_* is asymptotic length in an otolith scale, which is hypothetical otolith length at age of infinity; *k* is a growth coefficient; and *t*_0_ is hypothetical age when length equals zero. Three levels of hierarchy included measurements of annuli at age, nested in a fish individual, again nested in a group of a catch year. As a result, *L_∞_* and *k* parameters were estimated for each individual and also each catch year. We took the most conservative approach of priors, applying a gamma distribution for catch years and normal distribution for individuals with relaxed standard deviations (details in scripts in the provided GitHub repository, see Data and materials availability). To fit the model, 100,000 iterations were observed for three Markov chain Monte Carlo (MCMC) chains, and the first 10,000 were discarded as burn-in. The median of potential scale reduction factor $\hat{\left(R\right)}$ was 1.0036, and model convergence of the chains was visually examined in addition (fig. S11). As an additional assessment of the model, residuals were calculated from estimated otolith length from the model and observed length of otolith annuli (fig. S12). Here, the variance of residuals is larger for the first year that could be caused by the uneven number of observations that were fed to the model for each age. Nevertheless, the overall residuals remain near zero for all years. To avoid any bias of condition towards bigger fish, relative condition factor (*35*) was used to test whether fish condition could predict any of the growth parameters and Φ. Back-calculation of fish length was conducted using an equation from (*103*), using biological intercepts specific for Baltic cod (*104*). Accordingly, *L*_0_, which is the fish length at age 0, was set to 4.3 and *O*_0_, the otolith length at age 0 was set to 0.01.

### DNA extractions

For genetic materials, DNA was extracted using otoliths from earlier years (1996–1998, 2002, and 2008) and fin clips from recent years, 2014 and 2019. Otoliths and fin clips were always handled with tools (e.g., forceps), which were cleaned with ethanol 70% and sterilized in between each individual sample to avoid cross contamination. The extraction procedure for both otoliths and fin clips were conducted following the standard protocols from either the DNeasy Blood & Tissue Kit (QIAGEN, Aarhus, Denmark) or the NucleoSpin Tissue Kit (Macherey-Nagel, Düren, Germany). Otoliths were fully submerged in the lysis buffer to lysate any remnant tissues and then removed from the buffer. The lysate then was treated as in the manuals provided by the kits. Fin clips were cut into small pieces (up to 25 mg), submerged in a lysis buffer, and then continued following the protocols. The extracted DNA was purified using the QIAGEN QIAquick PCR Purification Kit (QIAGEN, Aarhus, Denmark). DNA quality was checked with standard electrophoresis in 1% agarose gel, and the quantity was measured using NanoDrop and Qubit Assay (Thermo Fisher Scientific, Carlsbad, USA).

To exclude samples suffering from cross-contamination (i.e., presence of tissue of several cod individuals in one otolith) that might have occurred during the sample collection, e.g., via insufficient cleaning of sampling tools since historic sampling was not intended for genetic analyses but rather for age readings, microsatellite (MSAT) analysis was done for DNA extracted from otolith samples. Four MSAT sites were used. A multiplex polymerase chain reaction (PCR) was conducted with four primer pairs on a 96-well plate. The PCR product was mixed with Hi-Di mix (Thermo Fisher Scientific, Applied Biosystems, Carlsbad, USA) with GeneScan LIZ dye Size Standard (Thermo Fisher Scientific, Applied Biosystems, Carlsbad, USA). Capillary electrophoresis was done with the reaction mix using ABI PRISM 3100 Genetic Analyzer (Thermo Fisher Scientific, Applied Biosystems, Carlsbad, USA). The MSAT peaks were analyzed using GeneMarker software (Softgenetics, State College, USA). As the chosen MSAT loci are very heterozygous (*H*_exp_ > 0.6) and typically display more than 10 alleles per site in a population, the likelihoods of encompassing overlapping alleles at any biallelic MSAT locus under sample contamination are small and virtually zero if several MSAT loci are combined, as is the case here. Thus, samples showing more than two allele peaks for any MSAT locus were identified as cross contaminated and subsequently excluded from the dataset (see examples in fig. S13).

### Library preparation and sequencing

Paired-end library preparation [2 × 100 base pairs (bp)] for 16 samples from 1996 was done in the Ancient DNA Laboratory at the Institute of Clinical Molecular Biology as a pilot to check if they should be treated specially like historic DNA samples. The details of the manual library preparation can be found in the method section in (*105*). For the fin clip samples from 2014 and 2019, 2 × 150-bp paired-end libraries were prepared using Illumina DNA Prep kit (Illumina, San Diego, USA) by the Competence Centre for Genomic Analysis (CCGA) Kiel. These libraries (16 otolith samples from 1996 from pilot and 40 fin clip samples from 2014 and 2019) were sequenced on Illumina 6000 S4 Flowcell (Illumina, San Diego, USA) by CCGA Kiel. In the end, it was concluded that older otolith samples can be treated the same as the rest, yielding sequence data of comparable quality. Thus, the rest of the samples, including phenotype samples from 1996 to 1998 and random samples of 1996, 2002, and 2008, were sent to Norwegian sequencing center for 2 × 150-bp library preparation using an Illumina Nextera DNA library preparation kit (Illumina, San Diego, USA) followed by sequencing on Illumina NovaSeq S4 Flowcell (Illumina, San Diego, USA).

### Read processing and variant calling

All sequenced reads from this study were processed together with published population data from (*40*), to include 23 EBC (named BOR), 22 WBC (KIE), and 24 North Sea (NOR) cod samples, which were later partitioned out. This was to identify WBC in our samples and test for any sequencing bias in our samples (fig. S9) as well as to conduct ancestry painting, which includes WBC and EBC individuals of known inversion status as reference (explained below). All sequenced reads were processed following the Genome Analysis Toolkit (GATK) best Practices workflow by Broad Institute (GATK v4.1.9.0) (*106*). All the detailed commands, parameters, and filtering options in the bioinformatics workflow are included in the provided GitHub repository (see Data and materials availability). Mapping to the reference genome of Atlantic cod, gadMor3.0 [National Center for Biotechnology Information (NCBI) accession ID: GCF_902167405.1], the median coverage of each individual ranged from 2× to 31× with a median of 12× for all samples. Two samples from 1996 were excluded on the basis of their low mapping coverage below 5×, as mapping coverage below 5× would compromise the accuracy of SNP calling (*107*, *108*).

After variant calling, raw SNP variants were first hard filtered on the basis of different qualities of variant sites according to best practices. Then, only biallelic SNPs were selected and filtered again on the basis of genotyping quality, missingness, read depths, and MAF of 0.005 to produce the final variant call file in a vcf format containing 5,847,389 variants for random samples. When possible, this full set of variants based on MAF > 0.005 were used, although some analyses were carried out using 4,685,343 variants filtered with MAF > 0.01, due to either the processing time or the resource limitation. Then, 679,584 variants with MAF > 0.05 among both random and phenotype samples were used for specific recommendations provided by analysis being applied.

Further analyses were done with two separate sets of variants resulting from different partitioning of the total sample set (also partitioned from WBC and North Sea samples), as parts of the sampling were intentionally biased for phenotype samples as explained earlier. (i) One hundred fifteen of random samples were used for the analysis identifying signatures of selection over time. (ii) A total of 152 samples including random and phenotype samples were used for genotype-phenotype association. The subset of the master vcf file was created using bcftools v1.2 (*109*), and, then, fixed sites were removed using GATK SelectVariants (v4.1.9.0).

### Population statistics and PCA

To examine any temporal differentiation in EBC independent of phenotypic data, 115 random samples were used to compute nucleotide diversity ( π ), between population nucleotide divergence (*d_xy_*), F_st_, and PCA. For calculating π and *d_xy_*, guides provided by Pixy (1.2.7.beta1) (*110*) were followed. A vcf file containing invariant sites was created, using GATK GenotypeGVCFs with option –all-sites followed by site filtering steps using GATK VariantFiltration with same criteria as in hard filtering of variants and followed by vcftools v0.1.16 (*111*) on missingness of 0.8 and mean read depths of 10. This filtered all-site file was combined with the final variant file to create the input vcf file for Pixy. A total of 81,462,138 records including invariant and variant sites, were used to calculate π for each catch year and pairwise *d_xy_* in 50-kb nonoverlapping windows. For genome-wide nucleotide diversity for each temporal population, average π value for all windows was calculated according to the equation provided by Pixy.

PCA on the subset of SNPs (4,685,343 after filtering for MAF > 0.01) was carried out using the R package pcadapt v4.3.3 (*112*). Scree plots of total variance explained by each PC were examined to decide up to which PCs to investigate. When all sites were included, a unique clustering pattern driven by inversion status of individuals appeared (fig. S14). Thus, sites within the inverted regions (identified as described below) were excluded then pruned on the basis of linkage disequilibrium (2,030,929 SNPs) to examine the remaining population structure.

Weir and Cockerham’s F_st_ was calculated using vcftools v0.1.16 in 20-kb windows. Only weighted F_st_ was used for plotting and interpretation of the data. All plots were created in R (R Development Core Team, 2022) using the base “plot” function.

### Genome-wide temporal covariance and simulation

Genome-wide temporal covariance was calculated using a modified python script in Jupyter notebook based on the functions in cvtkpy (http://github.com/vsbuffalo/cvtk) published in Buffalo and Coop (*37*). Error bars were calculated by bootstrapping covariance values, resampling blocks of loci 5000 times, using the bootstrap function provided by cvtkpy. As initial genome-wide temporal covariance showed an inconclusive pattern, we simulated a neutrally evolving population to compare the covariance values as a control. First, backward-in-time simulation was used to create a population with matching diversity using msprime v1.2 (*113*), with mutation rate of 3.5 × 10^−9^, recombination rate of 3.11 × 10^−8^, 5000 genomes, and a sequence length of 30 Mb. With this population as a founding population, a forward-in-time simulation was conducted using SLiM v2 (*114*). Additional 100 generations were burned in at the beginning of the simulated time. From generation 101, 20 individuals were sampled from the simulated population for five generations, to imitate the sampling scheme of wild population. Final vcf file was created to calculate the covariance of the simulated temporal populations. This was replicated 100 times to create a distribution of patterns from neutrally evolving populations. For the calculation of temporal covariance, a custom script in R language was used which replicated the functions in cvtkpy.

### GWA analysis

To identify specific genomic regions responsible for growth variation in the EBC population, GWA study was conducted. Growth performance was converted into an index using the growth estimates, Φ = logk+2 log L∞ (*34*). Subsequently, this variable was subjected to a univariate nonlinear mixed model to identify loci associated with the growth change using GEMMA v0.98.3 (*115*). A total of 679,584 SNPs were used after filtering for MAF of 0.05 and missingness of 0.1 as recommended by the developers. Genetic population structure was considered as a random effect and sex as covariates to incorporate and eliminate possible other contributing factors. Genomic inflation factors and QQ plots showed that systematic biases were adequately corrected from the other contributing factors (fig. S15). After correcting for multiple testing, using false discovery rate (*116*), with the number SNPs sites not in linkage disequilibrium (174,541), there were no individual SNP sites with genome-wide significance for Wald test *P* values observed. Instead, we used the GWA as intermediate step to enrich for loci that are likely to be associated with our target trait growth rate. To do so, we kept all SNP loci occupying the 0.05% tail of distribution of the *P* values, which resulted in 336 outliers for further downstream analysis (referred to as “GWA outliers”).

### Calculating and bootstrapping temporal autocovariance of GWA outliers

To demonstrate the directional changes over time in allele frequencies of the GWA outliers that are accountable for the growth variations, temporal covariance of the outlier loci was calculated in R. We used delta values of different time windows, lag-2 and lag-3, contrary to those with lag-1 provided in the cvtkpy package, which always uses consecutive time points to calculate the allele frequency changes. This was to avoid including a shared time point in calculating autocovariance, which showed positive covariance values in the simulated neutral populations and was likely driven by the shared time point rather than a true signal of selection. To assess the significance of observed covariance, a permutation test was conducted calculating temporal covariance values using 336 random loci sampled from all SNP sites in GWA analysis. The observed values were compared to the distribution of 1000 random permutations.

### Gene identification and GO term analysis

To further assess the biological relevance of any outlier loci or windows from genomic analysis, two approaches were used: (i) by searching for functional annotations in targeted genes for GWA outlier SNPs and (ii) by GO term enrichment analysis using a set of outliers. For (i), among the 336 SNPs assigned as GWA outliers, only regions with clustering outliers with flanking SNPs with low values (marked with red arrows in fig. S12) were examined in depth. Genes located at or within 5 kb up- and downstream of the outliers were further searched for their biological functions in the literature. The search was carried out using the gene names or descriptions, targeted with or without key words, e.g., fish, growth, maturity, and reproduction to find the most relevant functions to this study. Genes were listed by cross referencing each SNP to annotated genes in the gadMor3.0 annotation database (“gmorhua_gene_ensembl”) in Ensembl using the BioMart v2.54.1 R package (*117*). Same database and workflow were used in identifying genes lying within F_st_ outlier windows and in overlapping windows of F_st_ and GWA outliers. With the listed sets of genes, enriched GO terms were identified using the GO terms provided in the annotations of the gadMor3.0 database as “universe.” The workflow was based on the vignette provided by GOstats v2.64.0 R package (*118*).

### Identifying inversion status

Four large (5 to 17 Mbp) chromosomal inversions in Atlantic cod species have been previously identified (*19*), three of which are polymorphic in the EBC population. We targeted these regions as candidate supergenes that may have undergone selection over the study period and examined how their frequency changed over time. With prior knowledge of inversions located in LG2, LG7, and LG12, PCA was done on subset vcf files of each chromosome. Three distinct clusters of individuals of different inversion status [homozygous ancestral, homozygous derived, and heterozygous; “ancestral” status adopted from (*12*)] were observed, which was used for individual assignment. Then, F_st_ values were calculated among these three groups (each pairwise and global) and plotted to identify boundaries of the inversions (fig. S16). These boundaries were used to subset the bedfiles to feed as input of local PCA analysis. The inversion status of individuals was verified again by visually examining local PCA plots for each inversion status (fig. S17). When ambiguous, the individuals were visually examined for their genotypes in IGV v2.12.0 (*119*).

To identify the individual status of DC, ancestry painting was carried out following a tutorial from a GitHub repository of M. Matschiner (github.com/mmatschiner/tutorials/tree/master/analysis_of_introgression_with_snp_data). We used four samples (homozygotes ancestral, KIE1203003 and BOR1205002; and homozygote derived, KIE1202006 and KIE1203020) as reference of ancestral and derived homozygotes and two EBC [BOR1205003 and BOR1205007; identified in (*12*)] as “control” of DC. SNP sites between positions 6.5 and 7.5 Mb in LG12 [note that the location is different from that reported in (*12*) as different reference genomes were used], which are fixed 80% in these reference individuals, allowing for 20% of missingness, were painted two different colors in EBC individuals (fig. S8). DC status, either ancestral/derived homozygous or heterozygous, was assigned by visual examination.
