# Supplementary material for: Genomic evidence for fisheries-induced evolution in Eastern Baltic cod
Source: Sci Adv. 2025 Jun 25;11(26):eadr9889. doi: 10.1126/sciadv.adr9889 (PMC12189938; doi:10.1126/sciadv.adr9889)
Supplement: Supplementary file 1 — Figs. S1 to S17 Tables S1 to S4 [file sciadv.adr9889_sm.pdf]

Science Advances
AAAS

Supplementary Materials for

Genomic evidence for fisheries-induced evolution in Eastern Baltic cod

Kwi Young Han *et al.*

Corresponding author: Kwi Young Han, khan@geomar.de; Thorsten B. H. Reusch, treusch@geomar.de

Sci. Adv. **11**, eadr9889 (2025)
DOI: 10.1126/sciadv.adr9889

This PDF file includes:

Figs. S1 to S17
Tables S1 to S4

Figure S1. Boxplots of estimated individual von Bertalanffy growth parameters over time.

The individual level parameters were estimated in the growth model using "random" and "phenotype" samples. The posterior predictions of estimated parameters of individual level were grouped in temporal populations to calculate the median and the lower and higher whiskers. **(A)** L_∞, and **(B)** growth coefficient *k*, and **(C)** otolith radii at age 1 of all fish. Color codes are based on individuals' catch years as in the legend.

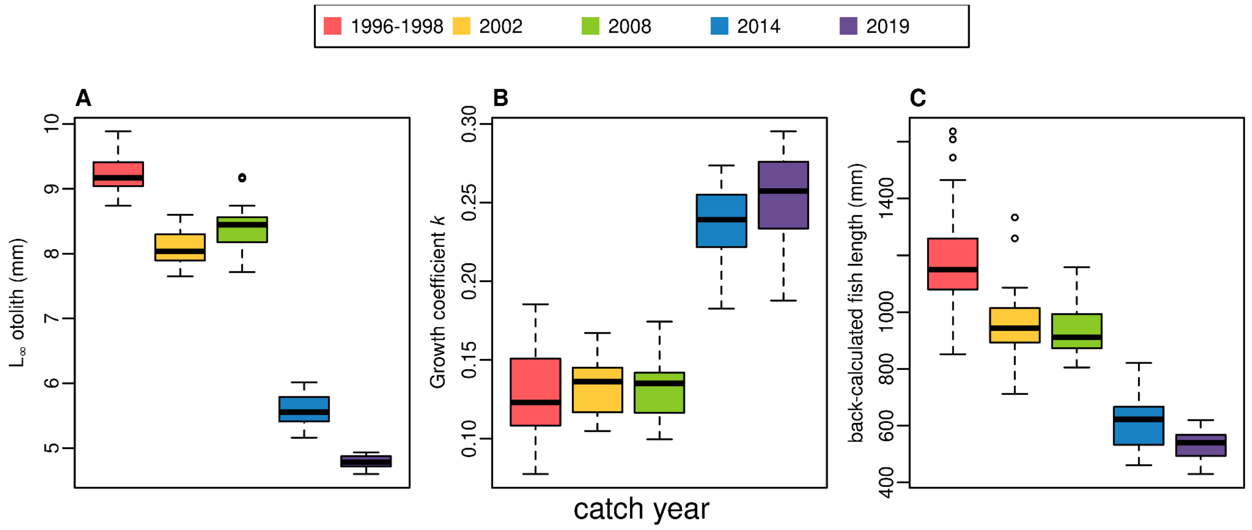

Figure S2. Fish condition at catch and relationship to growth.

Relative condition factor was calculated for individual fish using body weight and length at catch as potential predictors for individual growth. **(A)** Boxplot of condition for individual fish grouped based on the catch year, the temporal populations. Individual condition is plotted against (**B)** growth coefficient *k,* (**C**) asymptotic otolith length L_∞, and D. growth performance index, Φ. The correlation coefficients for each parameter are, r = -0.03 ($p > 0.05$), r = 0.09 ($p > 0.05$), and r = 0.09 ($p > 0.05$).

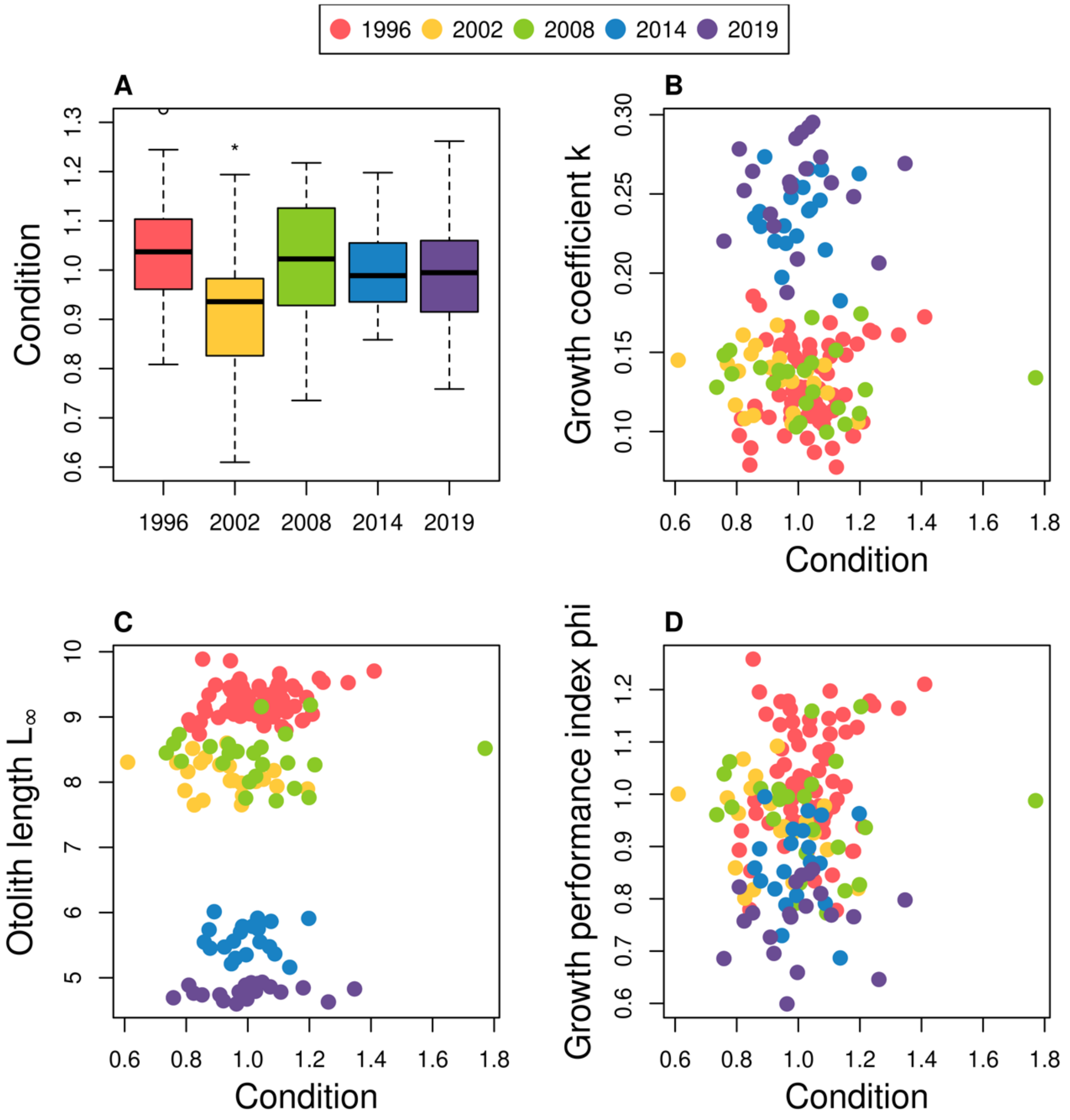

Figure S3. Genome-wide temporal covariance analysis on SNP polymorphism through time.

Each line in the covariance plot represents the temporal covariance, cov(Δp_s, Δp_t), calculated using allele frequency changes in two time windows of *s* and *t*; the lines are colored accordingly to rows of the temporal covariance matrix on the right side. The x-axis of the plot shows the later time windows in the calculation, Δp_t. For example, the three green points are, from left, cov(Δ1996-2002, Δ2002-2008), cov(Δ1996-2002, Δ2008-2014), and cov(Δ1996-2002, Δ2014-2019). Error bars (95% confidence interval) calculated by bootstrapping covariance values are drawn as lines over each point.

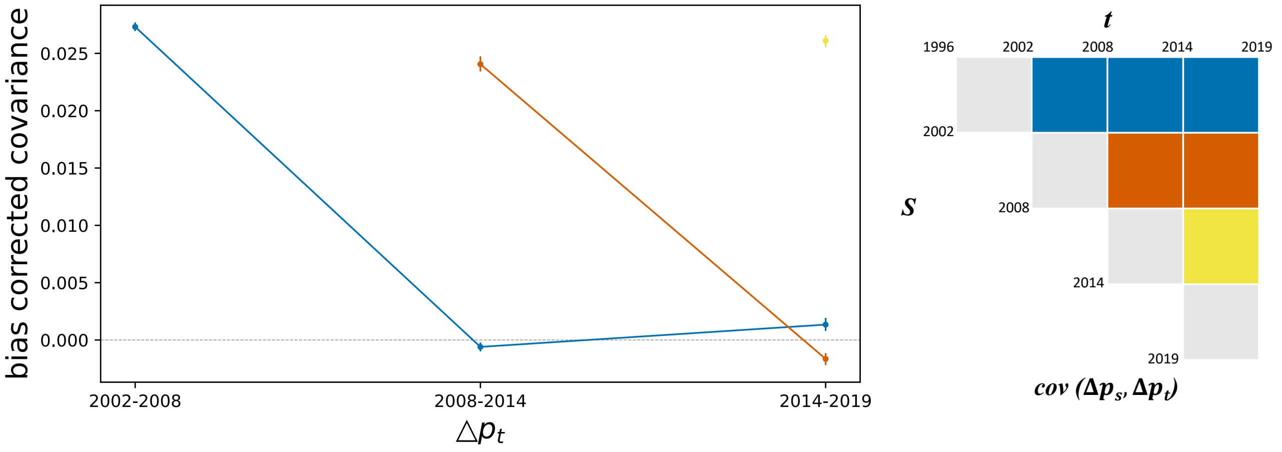

Figure S4. Simulated genome-wide temporal covariance through time in the absence of selection.

Similar to Figure S3, each line in the covariance plot represents the temporal covariance, cov(Δp_s, Δp_t), calculated using allele frequency changes in two time windows of *s* and *t*; the lines are colored accordingly to rows of the temporal covariance matrix on the right side. The x-axis of the plot shows the later time windows in the calculation, Δp_t. Instead of the catch years, simulated generations (t1 - t5) were used. The medians of all 100 simulated populations are plotted in colors and all temporal covariance values are plotted in grey lines to show random distribution of covariance values in a scenario of neutral evolution.

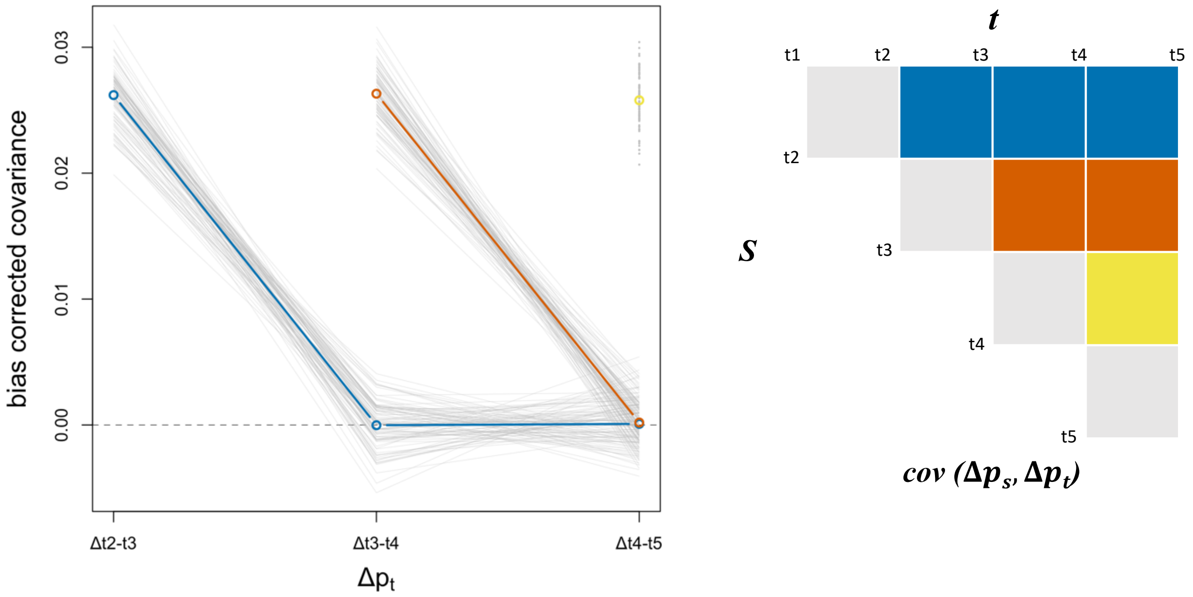

Figure S5. Distributions of the simulated temporal covariance values compared to covariance from real data in Figure S3.

To understand the significance of the genome-wide temporal covariance values, the values from real data (Figure S3) were compared to the simulated values (Figure S4). The histograms are frequency distribution of simulated values (100 replications) for each pairwise covariance of different time windows. The color codes follow the covariance matrix in Figure S4 of simulated time points (T1-T5), which corresponds to five time points 1996-2019. The red lines represent the covariance values of the real allele frequency changes for corresponding time windows. For example, for the first histogram of cov(ΔT2-T1, ΔT3-T2) were compared to cov(Δ2002-1996, Δ2008-2002).

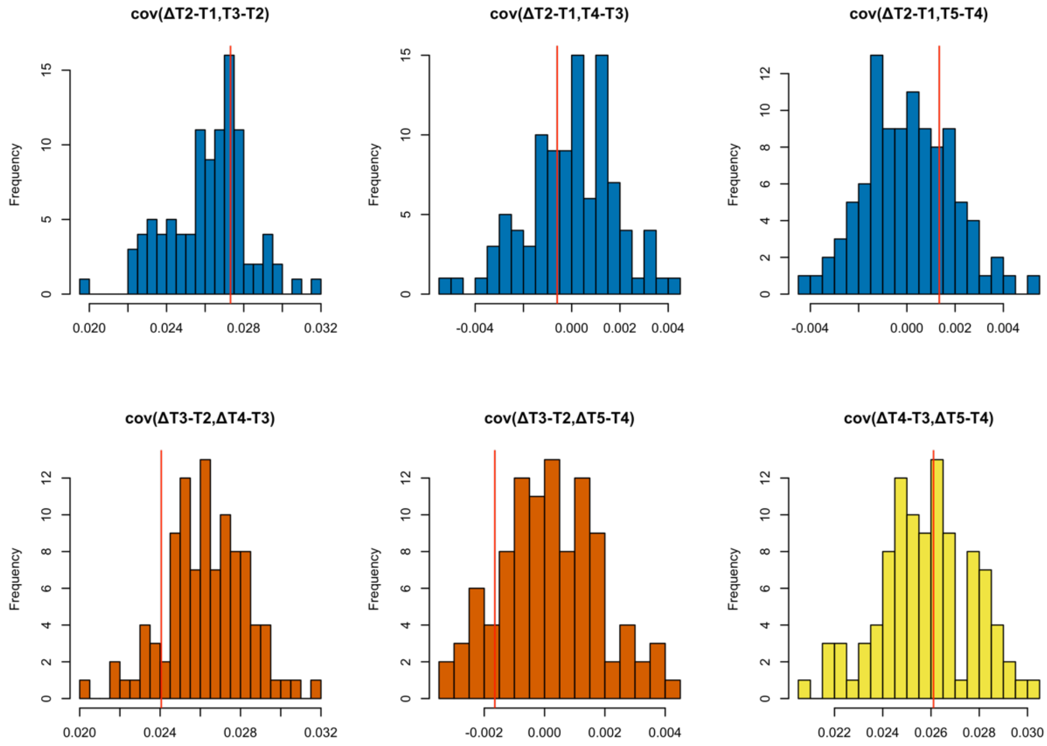

Figure S6. Population genetic statistics in 50Kb non-overlapping windows along the genome

(A) Nucleotide diversity (π) for each catch year and **(B)** absolute divergence between populations (d_{xy}) of first time point 1996 to all other years. Colors as in the legends above the plots. A total of 81,462,138 invariant sites and variant SNPs were used to calculate the values. For plotting, an R function "loess.smooth" was used to smooth the curves for visual ease.

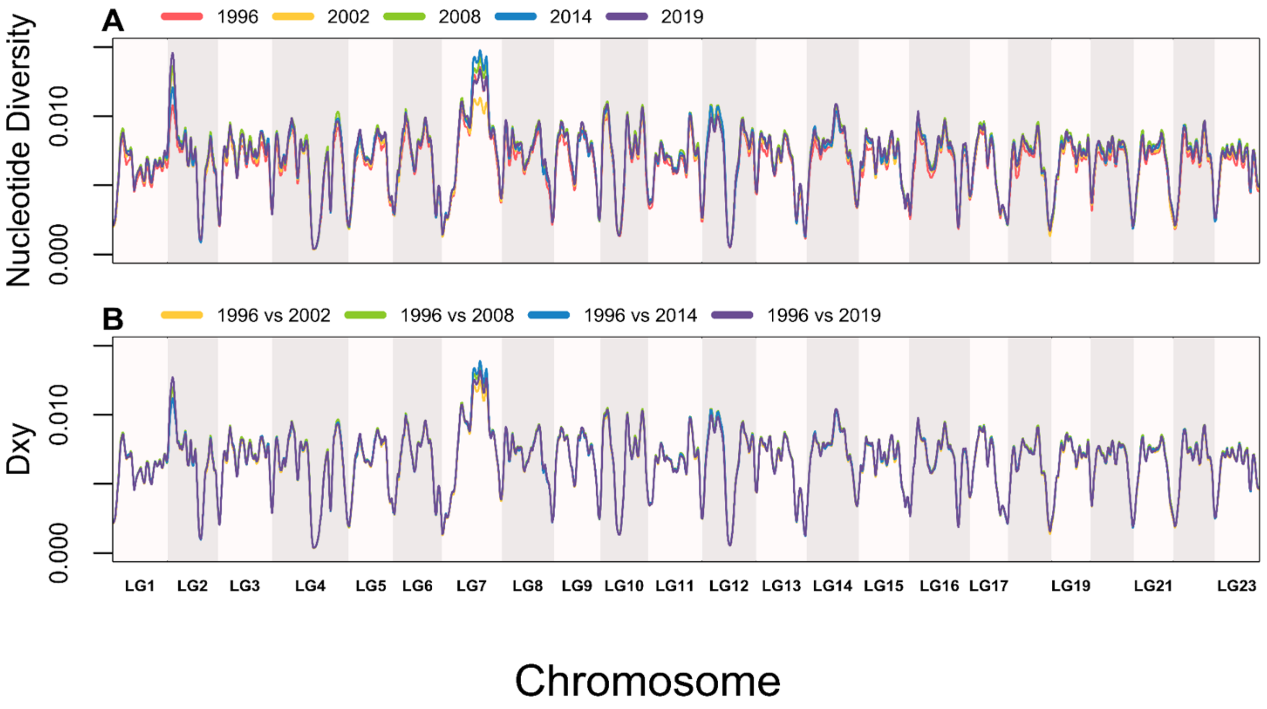

Figure S7. Frequency distribution of lag-2 and lag-3 temporal covariance of randomly permuted 336 SNPs compared to GWA outliers.

To detect signals of directional selection of GWA outliers which are correlated to growth performance, temporal covariance of allele frequency of the outliers were calculated and compared to values from randomly permuted 336 SNPs. The lag-2 and lag-3 time windows were used for calculating covariance to avoid shared time points, as in the inlets of each histogram visualized with a hypothetical allele frequency change. The frequency distribution of covariance of 1000 permutations of random SNPs are presented. The covariance was calculated in lag-2 time windows, namely cov(Δ1996-2008, Δ2002-2014) from top and cov(Δ2002-2014, Δ2008-2019), and lag-3 time window, cov(Δ1996-2014, Δ2002-2019) at the bottom. The red vertical solid lines depict the corresponding real covariance values of GWA outlier SNPs.

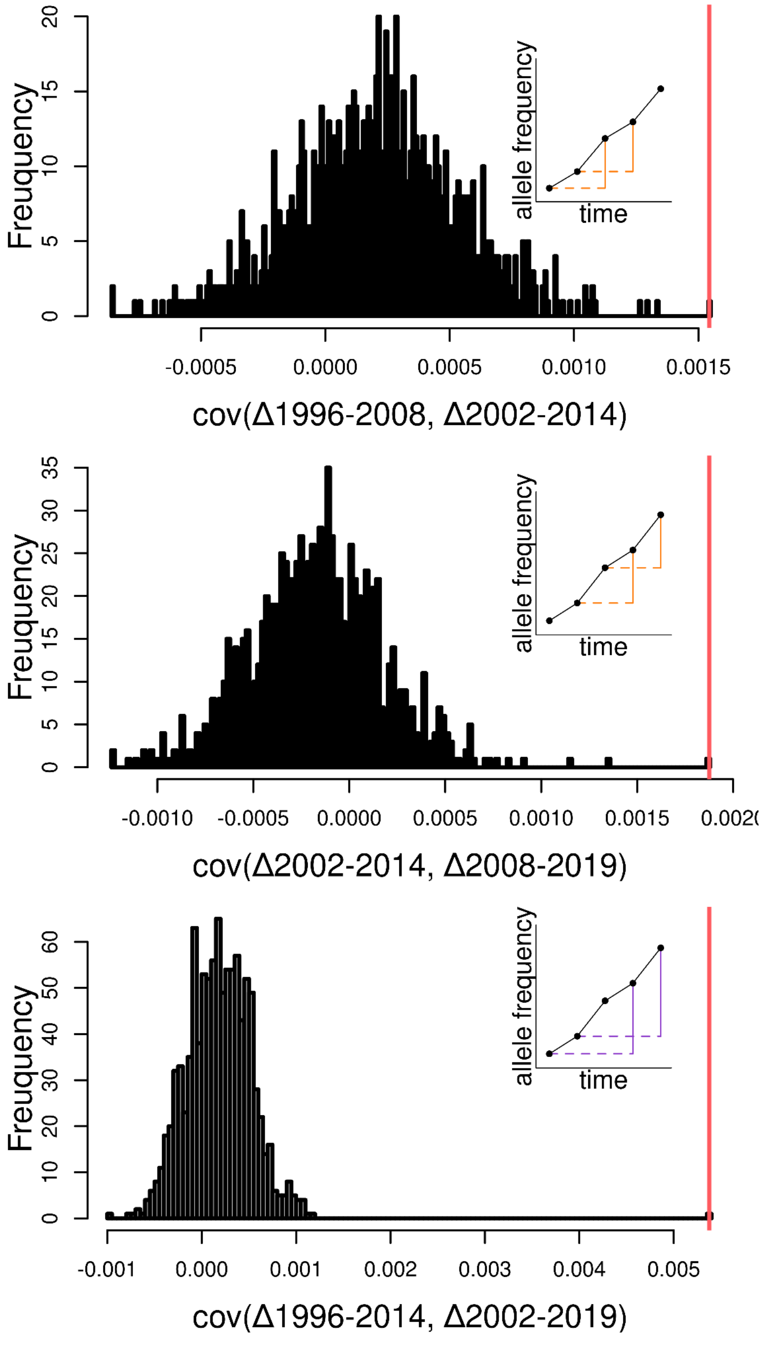

Figure S8. Ancestry painting for inversion in LG12.

To identify the double-crossover status of each individual, all "random" samples were examined by ancestry painting analysis, using four samples as reference of ancestral and derived homozygotes and two EBC as reference of double crossover. SNP sites between 6.5 and 7.5 Mb within LG12 (reference to garMor3.0) which are fixed 80% in two sets of references were painted depending on the allelic status of ancestral or derived (green and yellow respectively as in legend), allowing for 20% of missingness in the data. The double crossover region is visually identifiable with higher frequency of derived alleles (marked with red vertical lines across all samples). With two rows of genomes in each individual, the genotype status was assigned in three sections, before, at and after the double crossover region. Sample names in the plot correspond to the sequencing names in Table S1.

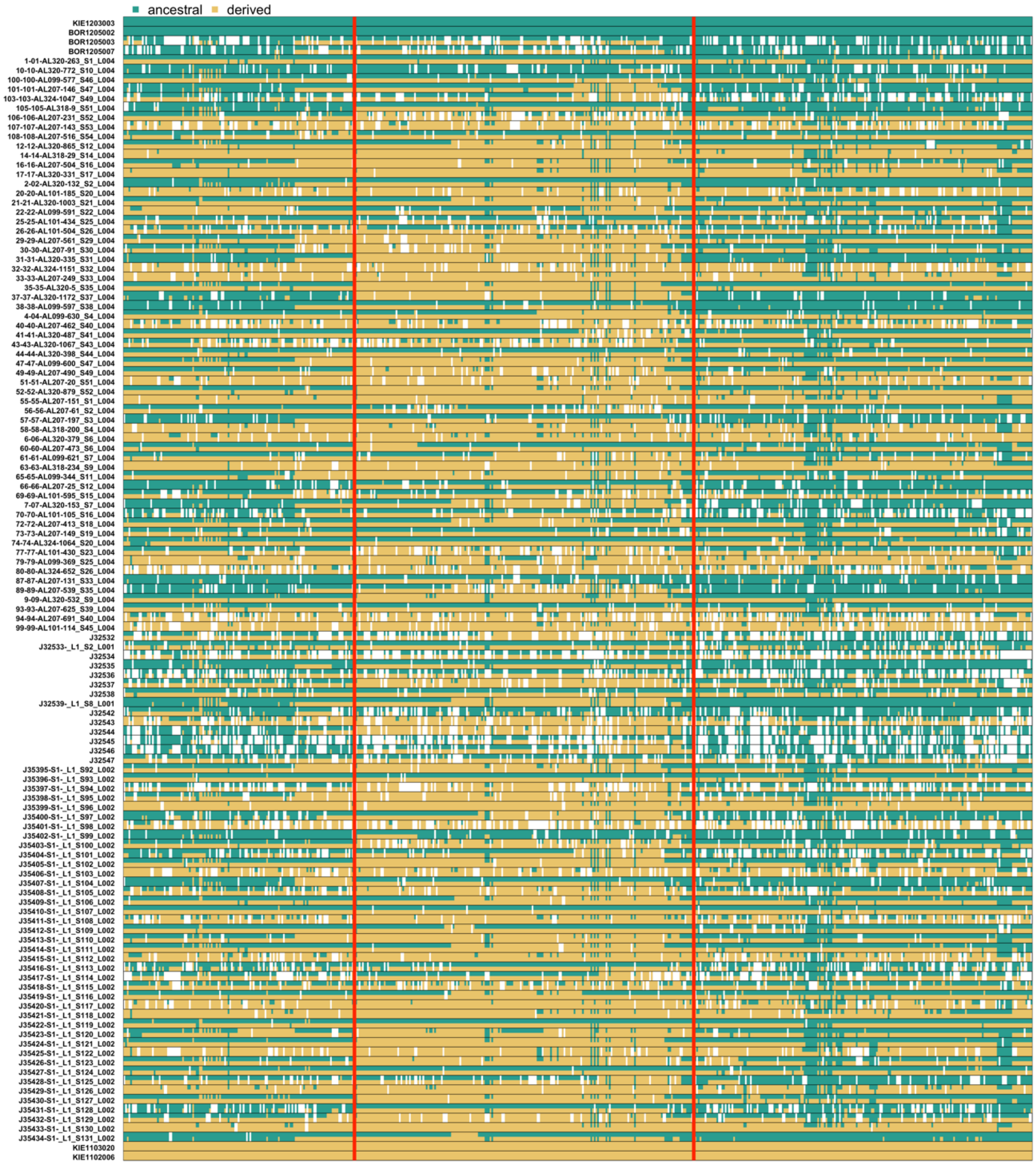

Figure S9. Principal component analysis including cod individuals from Barth et al. 2019 (40).

Principal component analysis was done with all sequenced samples from this study, “random” and “phenotype” together with other Atlantic cod populations of the Baltic and North Sea from Barth et al. 2019, 23 EBC (BOR); 22 WBC (KIE); and 24 North Sea (NOR). The goal was to identify potential migrants in our samples and test for bias in sequencing. Nine individuals of “phenotype” samples which cluster with WBC individuals were excluded in any downstream analysis.

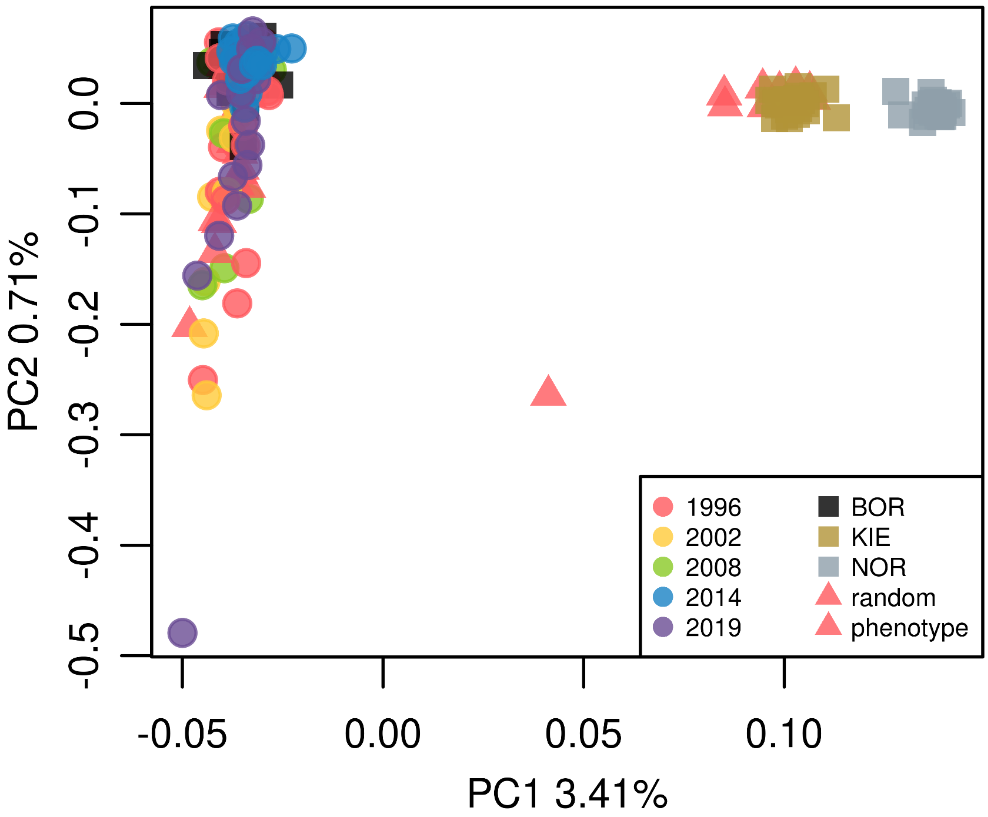

Figure S10. Element profiles of Phosphorus and Magnesium of sample used in this study.

Examples of life-long element concentration profiles for **A.** Phosophorus (P) and **B.** Magnesium (Mg). These profiles are from a 5-year-old female cod captured in July 1996 at a length of 57 cm and weight of 2275 g. The 4 blue dots and lines indicate the element minima detected using the standardized statistical approach. The red line indicates a minimum close to the edge that was not detected due to a limited number of data points. This was added to the count of minima to obtain the correct age of the fish.

A

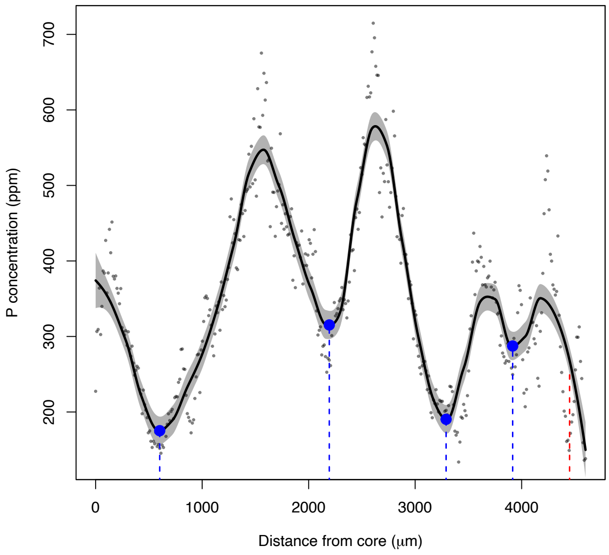

B

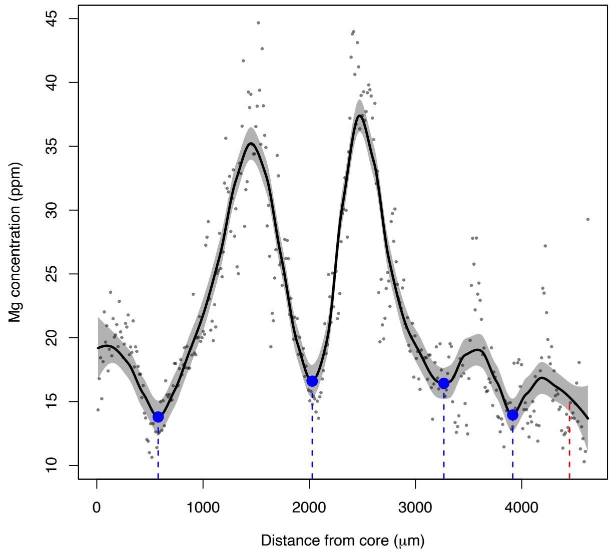

Figure S11. Posterior distributions and model convergence of three Markov chain analyses to estimate von Bertalanffy parameters.

The posterior distributions of four parameters used in the growth model (only three von growth parameters for all samples; *k.all, linf.all*, and *t0.all* and error *tol* are shown), in the left column, and corresponding trace plots of simulated three Markov chains over 100,000 iterations with a thinning rate of ten, in the right column, are plotted. Each chain starting from different initial values have converged adequately at the beginning of the iterations, indicating the robustness of model fit.

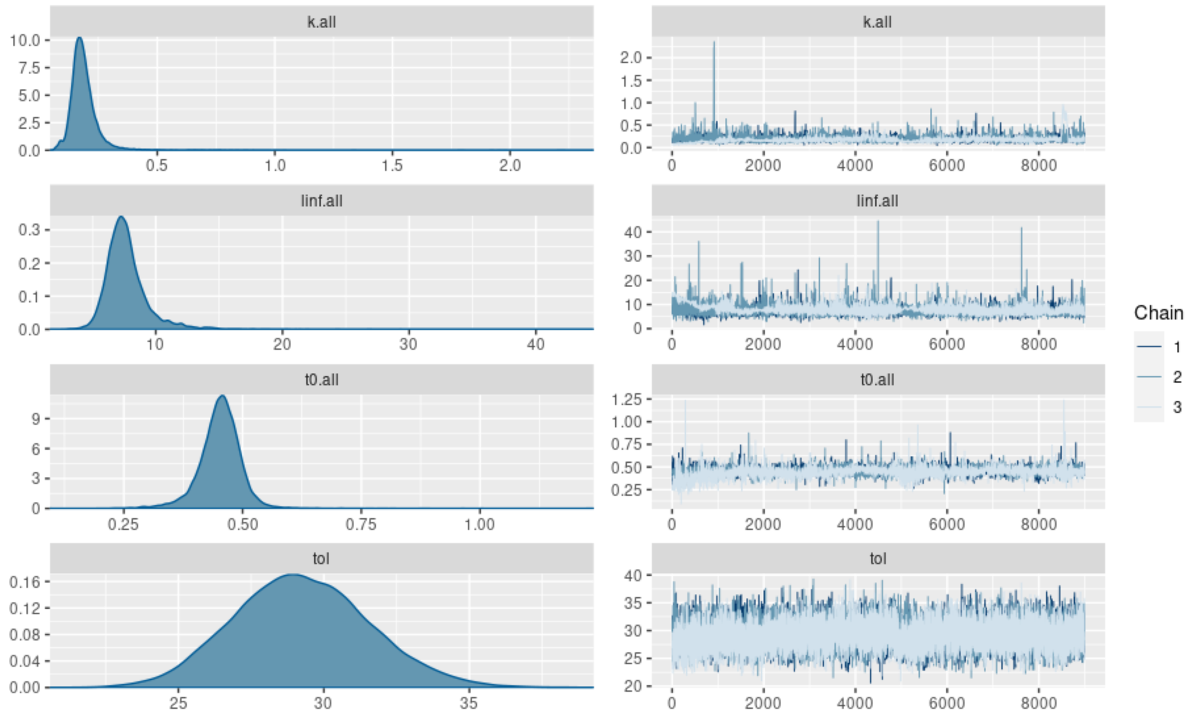

Figure S12. Residuals of estimated otolith lengths for each age class.

The residuals were calculated as (estimated otolith radii at age-observed otolith radii at age) / (observed otolith radii at age), matching the number of observations of each age class to assess the model fit. The discrepancy in variance of residuals in each age class is likely to come from the differences in the number of data points available in input (154 observations for age 1 and 1 observation for age 7).

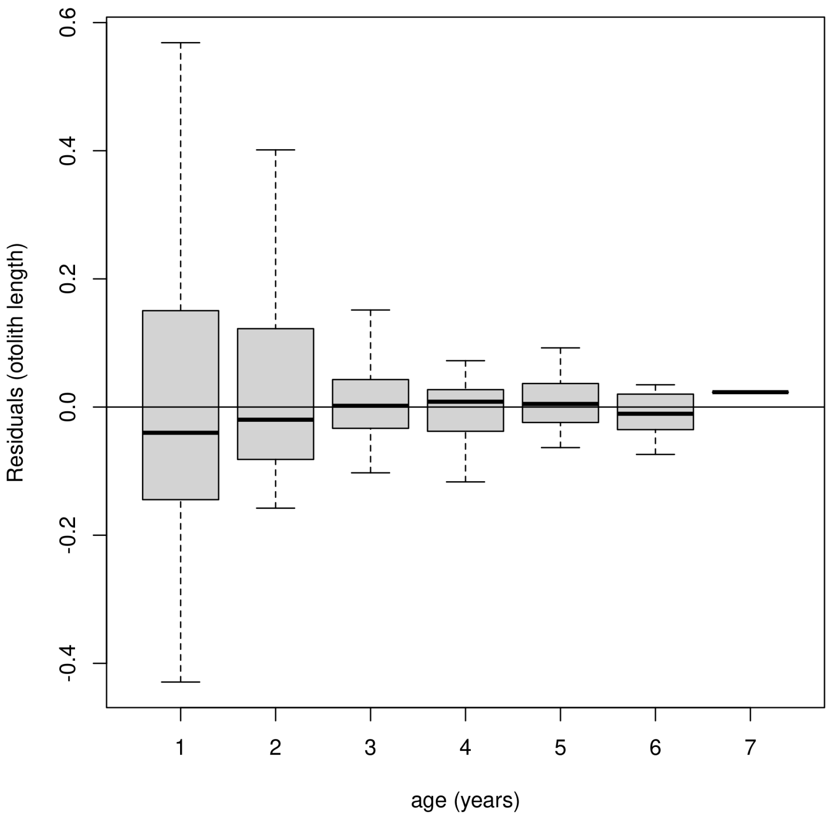

Figure S13. Segregation and electrophoretic visualisation of microsatellite (MSAT) alleles in clean and contaminated samples.

Four MSAT loci, (PGmo38 and Gmo5 blue; Tch11 black; GmoC18 red) were used to identify cross-contaminated samples. Prior knowledge of each MSAT length range in the resolution of 1bp was used to bin allele sizes using the GeneMarker® software. **(A)** An example MSAT panel of a clean sample. Each locus shows two clear peaks, indicating a heterozygous status, marked with grey vertical lines. For example, in PGmo38 (left side of blue panel), a peak at 95bp and the other at 105bp appear as two different alleles in the locus. The smaller peaks before the highest peak in each allele is a typical noise pattern from a characteristic of MSAT analysis, called "stutter band". The signal derives from incomplete products of multiplex PCR cycles, thus always smaller than the signal from complete products. **(B)** An example of cross-contaminated samples. When two (or more) individuals with different MSAT genotypes are inadvertently mixed in the DNA samples, three or more peaks appear as shown. For example, in Gmo5 (right side of the blue panel), three peaks are identified. As stutter bands are always smaller and increasing towards the real peaks, the last peak at size 195 bp (marked in red box) is likely to be a real signal of an additional allele. The other MSAT loci also show more than three peaks even after considering stutter patterns. All MSAT peaks of all individuals were examined manually to confirm and edit the automatic peak detection of the software. Samples with more than three peaks in at least one MSAT locus were excluded in the selection process.

A

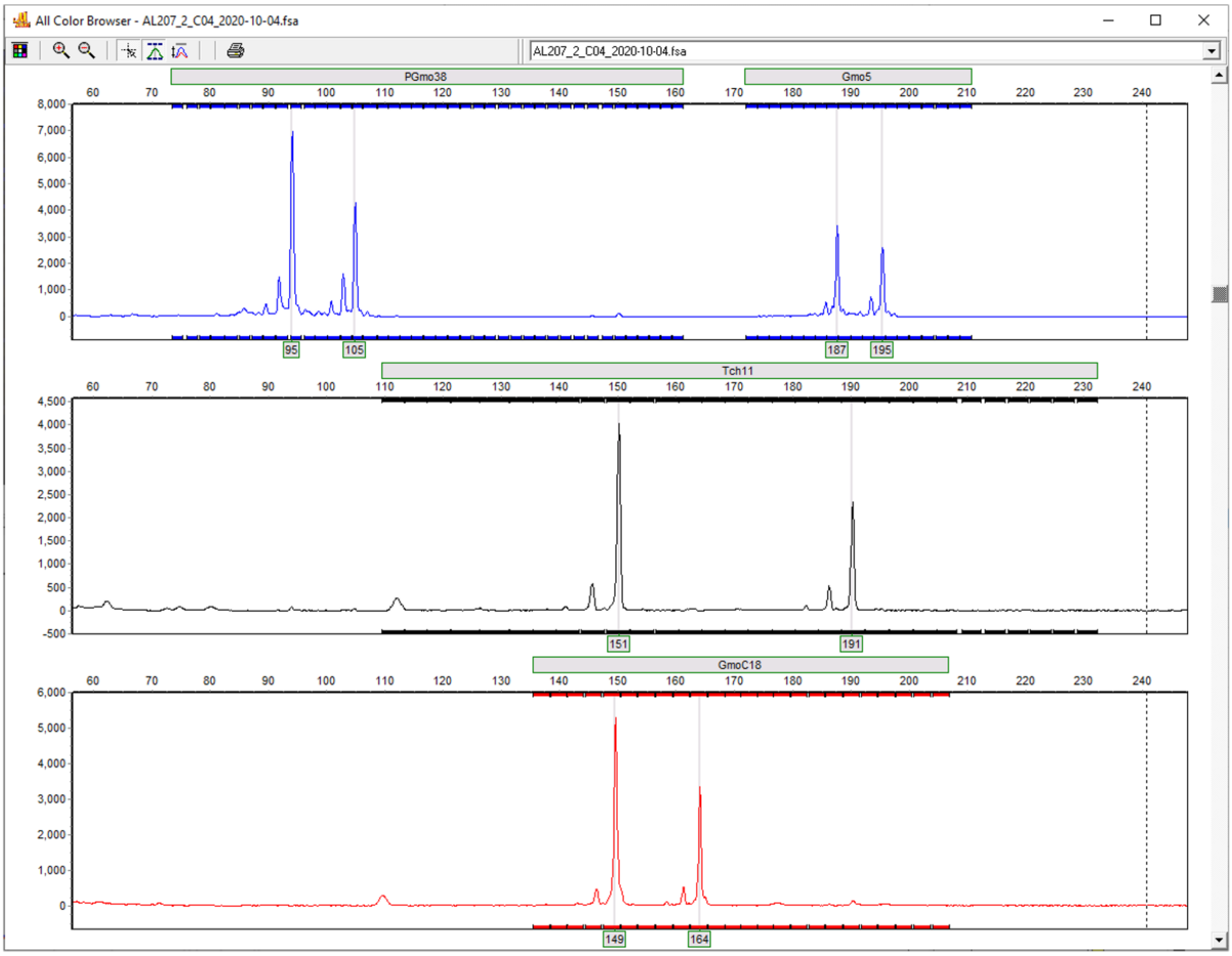

B

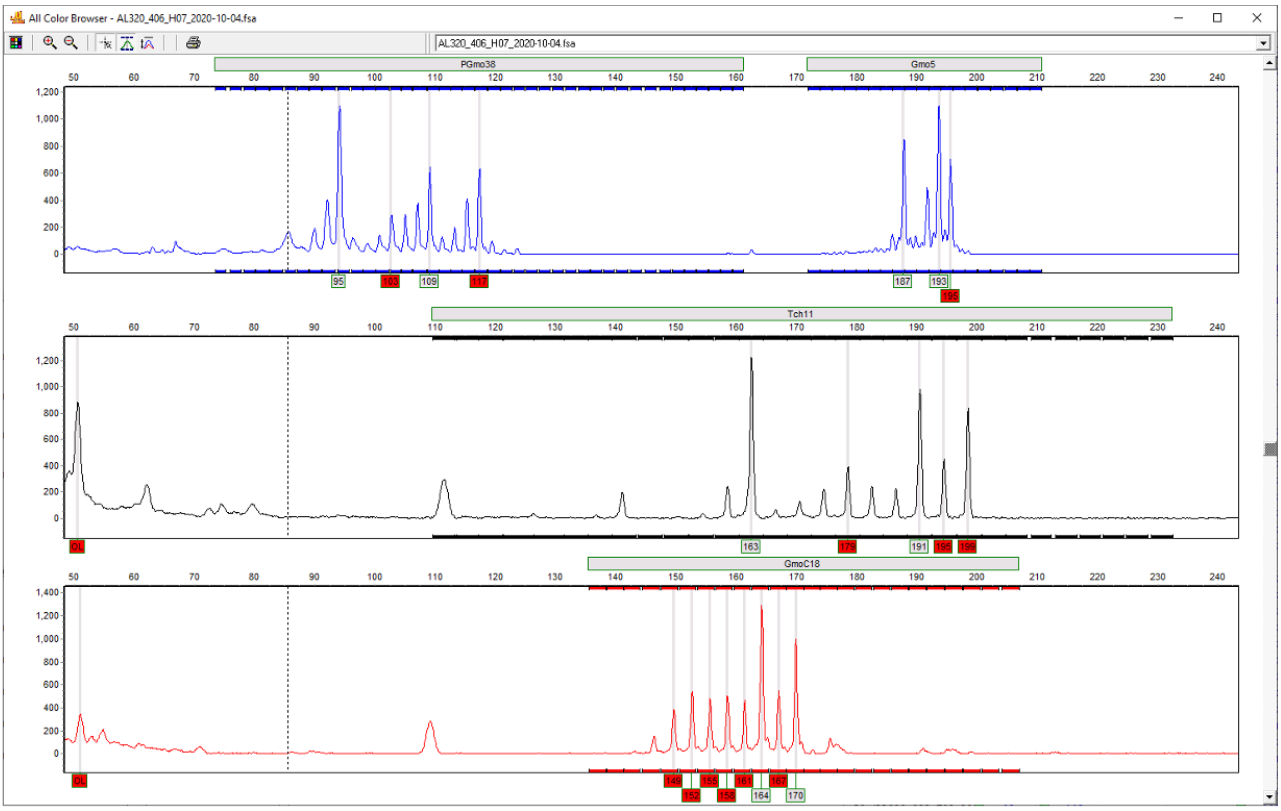

All Color Browser - AL320_406_H07_2020-10-04.fsa
AL320_406_H07_2020-10-04.fsa
PGmo38
Gmo5
95
103
109
117
187
193
195
Tch11
OL
163
179
191
195
199
GmoC18
OL
149
152
155
158
161
164
167
170

Figure S14. Principal component analysis of 115 "random" samples.

PCA was done with all variants of 4,685,343 SNPs (after filtering for MAF > 0.01). (**A)** PC1 explains 2.25% of all variations in the genotypes and PC2 explains 1.57%. The clustering pattern observed disappears in Figure 1D once the SNPs within inverted regions in LG2, 7, and 12 are removed**. (B)** Population structure on PC3 and PC4. Each individual is coded in color according to the catch.

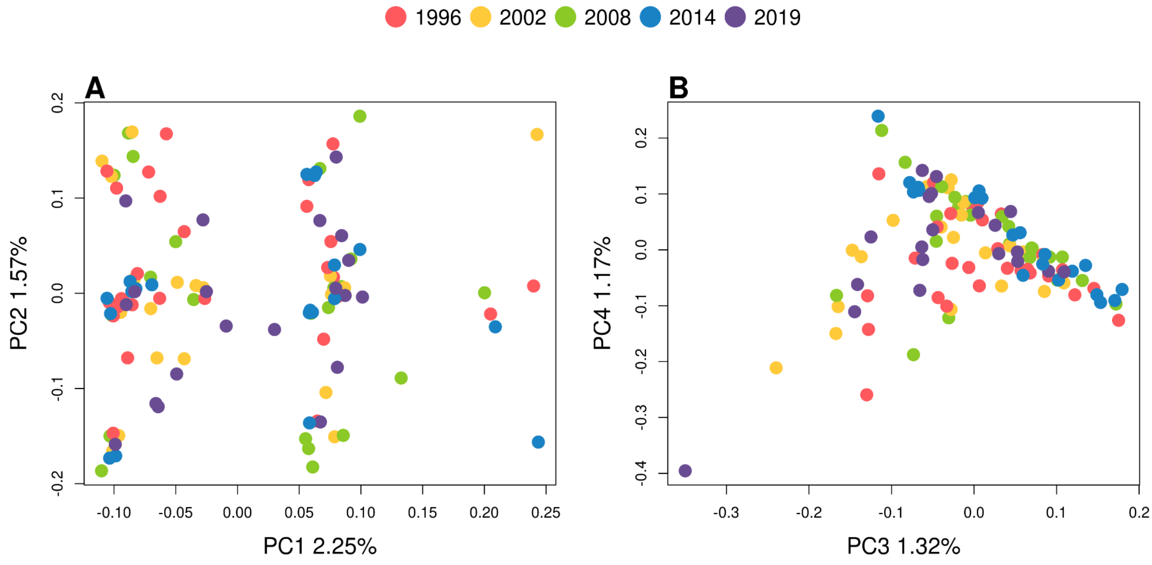

Figure S15. QQ plot of expected and observed chi-squared p-values of the GWA.

The observed chi-squared p-values of the GWA were plotted against chi-squared expected p-value distribution in a quantile-quantile (QQ) plot. The genomic inflation factor (= lambda), i.e., the median of chi-squared observed p-value divided by expected median of chi-squared p-value, was calculated. The overall fit of the expected versus the observed with a slight deviation at the highest end indicates an adequate correction of confounding factors during the GWA analysis.

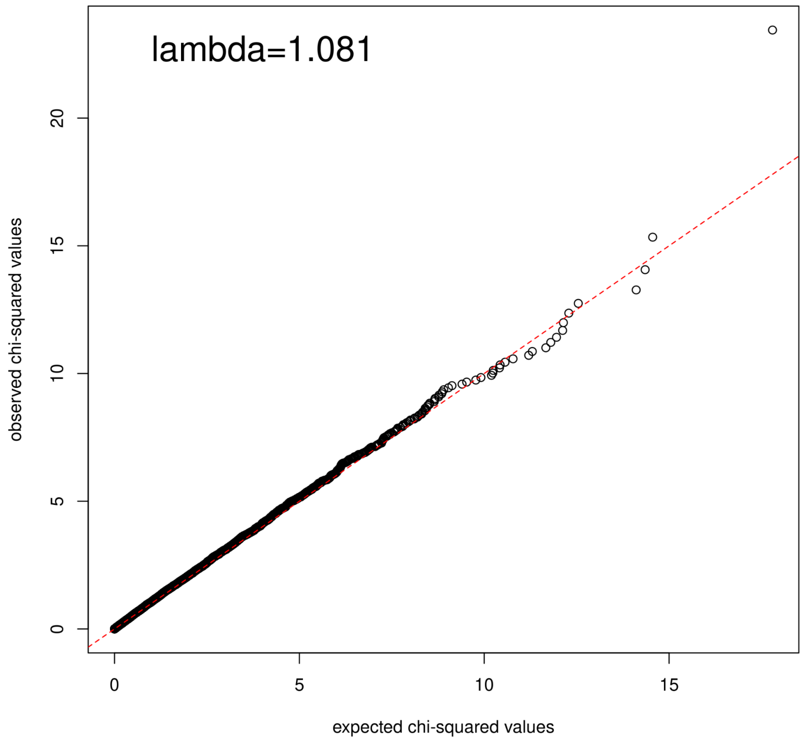

Figure S16. Global and pairwise F_{st} on groups of individuals based on the inversion status (LG2, LG7, and LG12).

To determine the boundaries of inversion referenced to the reference genome gadMor3.0, F_{st} values in 30Kb overlapping windows (in 15Kb steps) were computed using groups of individuals of different inversion status**.** After plotting the global F_{st} values over the whole chromosome (row 1), approximate coordinates were chosen to zoom in (row 2) to decide the beginning and end of the inverted regions (row 3 and 5). The pairwise F_{st} of two groups of homozygotes (ancestral and derived status) were also investigated to confirm the consistency of signals (row 4 and 6). Two locations were visually selected in the beginning and the end of inversions (Dashed vertical lines in row 3-6) to either include or exclude the regions depending on the downstream analysis conducted.

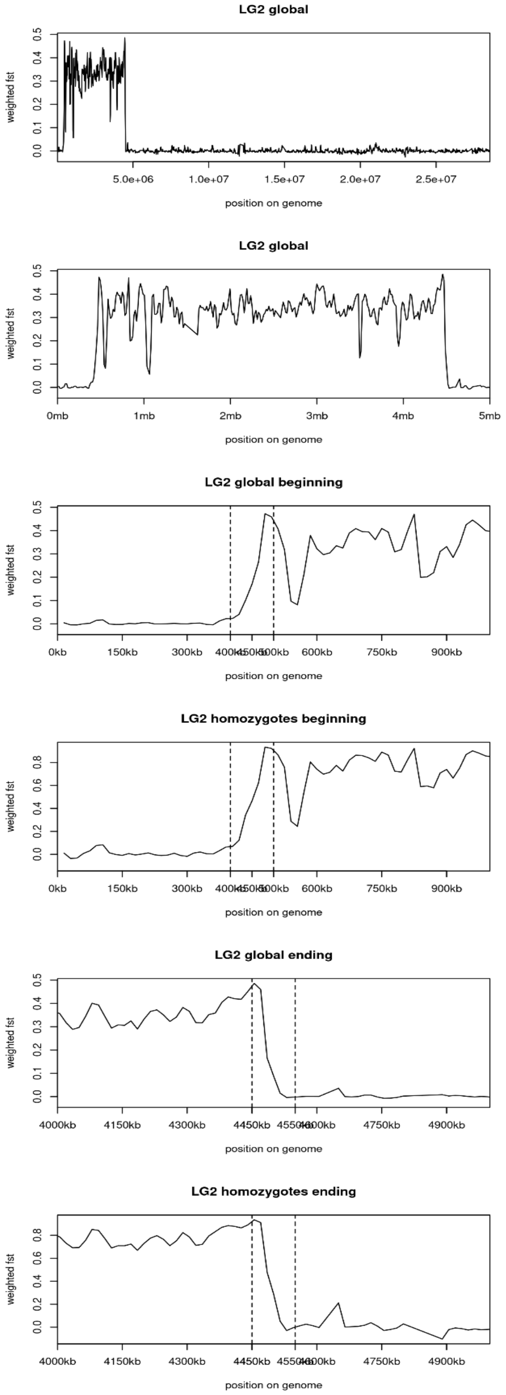

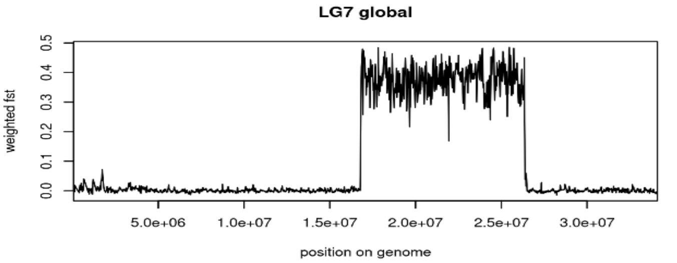
LG7 global
weighted fst
0.0
0.1
0.2
0.3
0.4
0.5
5.0e+06
1.0e+07
1.5e+07
2.0e+07
2.5e+07
3.0e+07
position on genome

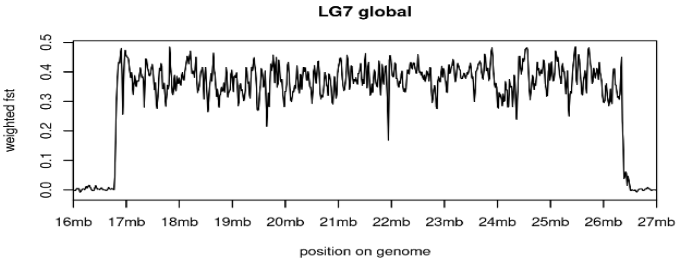
LG7 global
weighted fst
0.0
0.1
0.2
0.3
0.4
0.5
16mb
17mb
18mb
19mb
20mb
21mb
22mb
23mb
24mb
25mb
26mb
27mb
position on genome

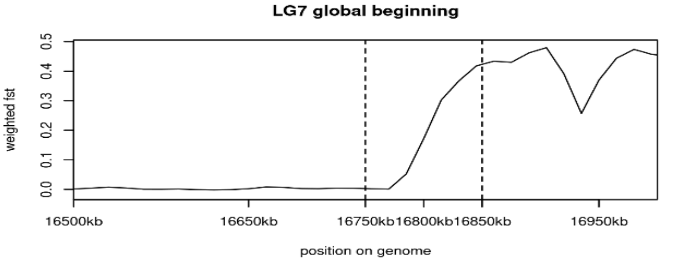
LG7 global beginning
weighted fst
0.0
0.1
0.2
0.3
0.4
0.5
16500kb
16650kb
16750kb
16800kb
16850kb
16950kb
position on genome

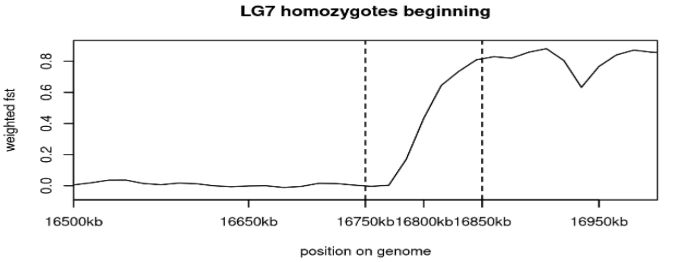
LG7 homozygotes beginning
weighted fst
0.0
0.2
0.4
0.6
0.8
16500kb
16650kb
16750kb
16800kb
16850kb
16950kb
position on genome

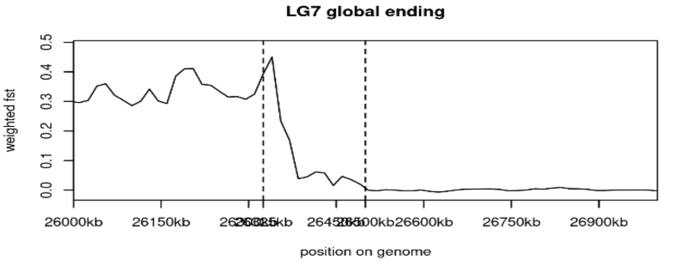
LG7 global ending
weighted fst
0.0
0.1
0.2
0.3
0.4
0.5
26000kb
26150kb
26600kb
26750kb
26900kb
position on genome

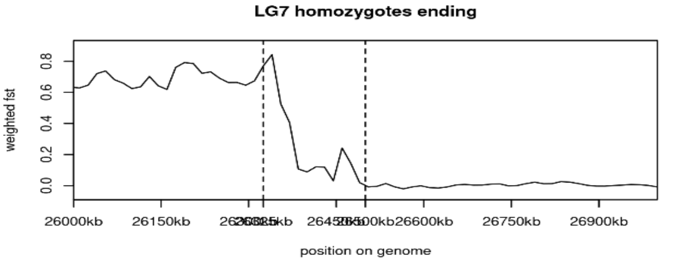
LG7 homozygotes ending
weighted fst
0.0
0.2
0.4
0.6
0.8
26000kb
26150kb
26600kb
26750kb
26900kb
position on genome

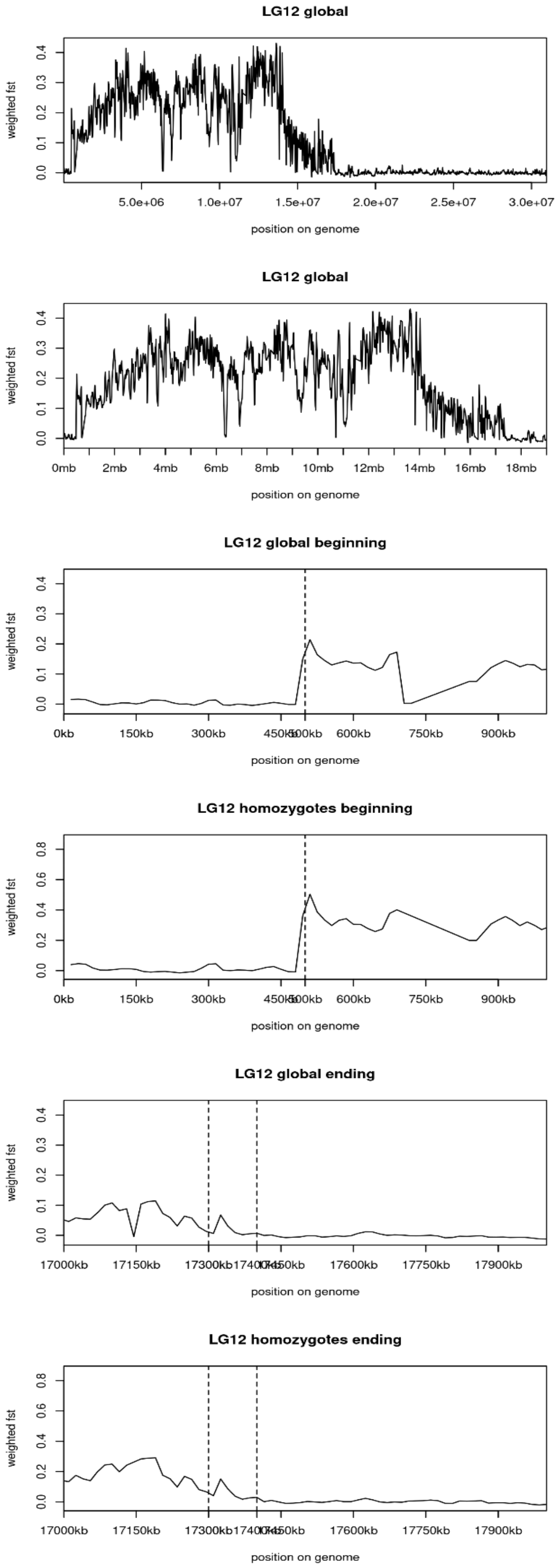

LG12 global
weighted fst
0.0 0.1 0.2 0.3 0.4
5.0e+06 1.0e+07 1.5e+07 2.0e+07 2.5e+07 3.0e+07
position on genome
LG12 global
weighted fst
0.0 0.1 0.2 0.3 0.4
0mb 2mb 4mb 6mb 8mb 10mb 12mb 14mb 16mb 18mb
position on genome
LG12 global beginning
weighted fst
0.0 0.1 0.2 0.3 0.4
0kb 150kb 300kb 450kb 500kb 600kb 750kb 900kb
position on genome
LG12 homozygotes beginning
weighted fst
0.0 0.2 0.4 0.6 0.8
0kb 150kb 300kb 450kb 500kb 600kb 750kb 900kb
position on genome
LG12 global ending
weighted fst
0.0 0.1 0.2 0.3 0.4
17000kb 17150kb 17300kb 17400kb 17450kb 17600kb 17750kb 17900kb
position on genome
LG12 homozygotes ending
weighted fst
0.0 0.2 0.4 0.6 0.8
17000kb 17150kb 17300kb 17400kb 17450kb 17600kb 17750kb 17900kb
position on genome

Figure S17. Local principal component analysis on inverted chromosomal regions in LG02, LG07, and LG12 of the cod Gadus morhua genome.

Local PCA was conducted using SNP sites within the inverted regions to identify individual inversion status. Three groups depending on the genotypes, ancestral and derived homozygotes on the sides and heterozygotes in the middle, are strongly clustered. Samples were assigned their status according to these clusters to calculate inversion frequency over time. Individuals are colored according to the catch year.

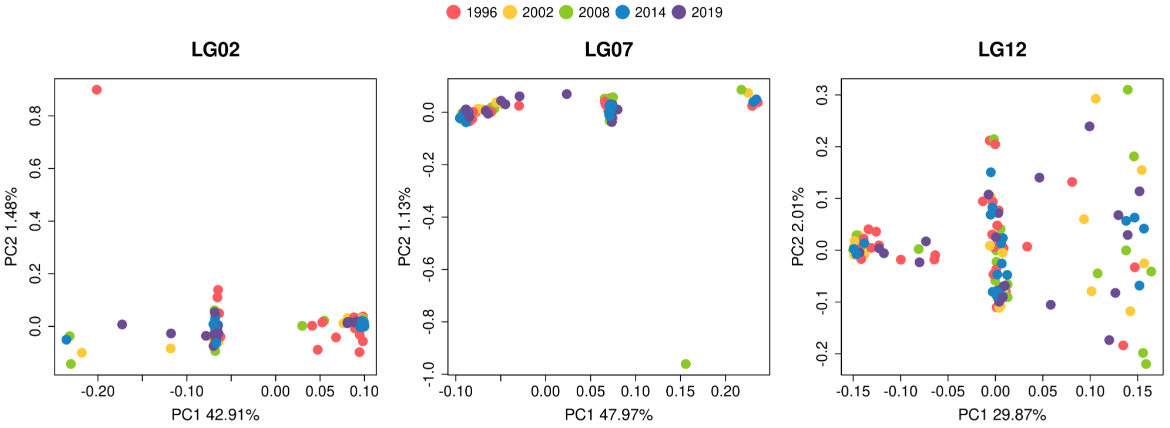

Table S1. All samples used in this study and metadata.

A total of 154 samples were subjected to sequencing and growth modelling. Two of them were excluded in any genetic analysis due to their low sequencing depth. "Otolith radius in mm" : the length from otolith core to the edge, "distance_min_x" : the distance from the core to the chemical annuli (minima) for each age estimate, "chemAge" : the estimated age from the age reading protocol, "sample.type" : the assignment of the sample type "random" and "phenotype" according to the sampling design. The samples were grouped into "bins" according to the catch year for growth modelling. The "phenotype" samples from 1996-1998 were grouped together with "random" samples from 1996 in "bin1". For all the genetic analysis, "SequencingName" were used and matched with "fishID" for metadata. This table is also available in the github repository.

fishID	catch.year	length in mm	sex	maturity	otolith radius in mm	chemAge	distance_min_1	distance_min_2	distance_min_3	distance_min_4	distance_min_5	distance_min_6	distance_min_7	sample.type	bins	SequencingName
AL097_334	1996	420	F	1	3.211704	3	0.7882197	1.9999617	3.10577	NA	NA	NA	NA	pheno	bin1	104-104-AL097-334_S50_L004
AL097_430	1996	260	M	4	2.3528964	3	0.4396807879	1.319046909	2.270547	NA	NA	NA	NA	pheno	bin1	75-75-AL097-430_S21_L004
AL097_552	1996	410	M	1	3.30582	4	0.6611660184	1.385672	2.231428877	2.786335818	NA	NA	NA	pheno	bin1	83-83-AL097-552_S29_L004
AL099_189	1996	460	F	1	4.317564	7	0.8161973752	1.466789481	2.259326806	2.850775876	3.31210568	NA	NA	pheno	bin1	39-39-AL099-189_S39_L004
AL099_326	1996	270	M	5	2.1646641	3	0.4941093	1.423503	2.01172563	NA	NA	NA	NA	pheno	bin1	64-64-AL099-326_S10_L004
AL099_328	1996	400	F	1	2.9293566	3	0.5646945	2.1999582	2.70583023	NA	NA	NA	NA	pheno	bin1	102-102-AL099-328_S48_L004
AL099_344	1996	310	M	5	2.5175976	3	0.399993	1.2470361	2.35289643	NA	NA	NA	NA	random	bin1	65-65-AL099-344_S11_L004
AL099_35	1996	890	F	6	5.450875	5	0.49018495	1.39213	2.86269	4.27443	5.058725	NA	NA	random	bin1	J32532-_L1_S1_L002
AL099_37	1996	1040	F	6	5.929299	6	0.70587	2.776419	3.6587559	4.5410901	5.1251896	5.7754189	NA	random	bin1	J32533-_L1_S2_L001
AL099_577	1996	570	F	6	3.835218	3	0.7005473147	2.64784067	3.775258	NA	NA	NA	NA	random	bin1	100-100-AL099-577_S46_L004
AL099_591	1996	350	M	7	2.541129	4	0.4845875811	1.004636149	1.678331314	2.475258	NA	NA	NA	random	bin1	22-22-AL099-591_S22_L004
AL099_597	1996	530	M	6	4.258746	4	1.047039	2.294076	3.5411103	4.185647	NA	NA	NA	random	bin1	38-38-AL099-597_S38_L004
AL099_600	1996	340	M	6	3.199941	4	0.329406	1.2588003	2.105844	2.847006	NA	NA	NA	random	bin1	47-47-AL099-600_S47_L004

AL099_602	1996	270	M	6	2.4705438	3	0.7529295	1.2352734	1.5293817	NA	NA	NA	NA	pheno	bin1	53-53-AL099-602_S53_L004
AL099_613	1996	580	F	6	3.92934	3	0.623517	2.4705417	3.450258	NA	NA	NA	NA	random	bin1	J32535-_L1_S3_L002
AL099_621	1996	630	M	7	4.599915	6	0.4138730706	1.064247408	2.38273266	3.068580604	3.783991621	4.245165247	NA	random	bin1	61-61-AL099-621_S7_L004
AL099_630	1996	430	M	6	3.517578	3	0.5665854198	1.085960329	2.679497306	NA	NA	NA	NA	random	bin1	4-04-AL099-630_S4_L004
AL099_636	1996	620	M	7	4.8430445	5	0.6666505	1.1764455	2.1372095	3.1450465	4.0783495	NA	NA	random	bin1	J32536-_L1_S4_L002
AL099_78	1996	560	M	6	4.411681	6	0.45097045	1.1176255	1.68624	2.4901455	3.313661	3.7058095	NA	random	bin1	J32534-_L1_S2_L002
AL101_105	1996	570	F	7	4.623444	5	0.6015187318	2.193776675	3.290665614	3.915776082	4.550167	NA	NA	random	bin1	70-70-AL101-105_S16_L004
AL101_114	1996	580	F	7	4.599912	6	0.823515	1.435269	2.141136	2.8411237	3.646989	4.5	NA	random	bin1	99-99-AL101-114_S45_L004
AL101_185	1996	590	M	6	4.282269	6	0.4507659285	1.328570843	2.052166361	2.882524952	3.6298459	4.2	NA	random	bin1	20-20-AL101-185_S20_L004
AL101_214	1996	540	F	8	3.164646	4	0.6978097159	1.564543397	2.323111806	3.1	NA	NA	NA	random	bin1	J32537-_L1_S5_L002
AL101_287	1996	460	M	7	3.682284	4	0.482346	0.988218	2.5058343	3.6	NA	NA	NA	random	bin1	J32538-_L1_S6_L002
AL101_288	1996	550	F	6	4.388151	7	0.5946012489	1.117850348	1.95028967	2.473538466	3.24652009	3.853009118	4.102741642	random	bin1	J32539-_L1_S8_L001
AL101_344	1996	430	F	7	3.670518	4	1.1411547	1.741143	2.8352406	3.6	NA	NA	NA	random	bin1	J32542-_L1_S9_L002
AL101_422	1996	560	F	7	3.905811	5	0.835278	1.470564	2.1764283	2.7528929	3.71757858	NA	NA	random	bin1	J32543-_L1_S10_L002
AL101_423	1996	480	F	7	3.435228	3	0.8470425	1.8940806	3.24699558	NA	NA	NA	NA	random	bin1	J32544-_L1_S11_L002
AL101_430	1996	540	F	7	4.25874	6	0.5323416666	1.005538617	1.940091808	2.489621822	3.111246793	3.927502105	NA	random	bin1	77-77-AL101-430_S23_L004
AL101_434	1996	430	M	7	2.9175936	5	0.462548792	1.079270837	1.897623088	2.039940722	2.407611293	NA	NA	random	bin1	25-25-AL101-434_S25_L004
AL101_442	1996	800	F	7	5.305779	6	0.6735619839	1.890701522	2.812416969	3.521431169	3.94683969	4.774019609	NA	random	bin1	J32545-_L1_S12_L002
AL101_504	1996	460	F	7	3.70581	4	0.5701255101	1.045227073	2.316130644	3.539523029	NA	NA	NA	random	bin1	26-26-AL101-504_S26_L004
AL101_523	1996	650	F	7	4.599912	5	0.6148993755	1.288922183	2.636967799	3.748516972	4.4	NA	NA	random	bin1	J32546-_L1_S13_L002
AL101_546	1996	500	M	1	3.449085	4	0.6470439	1.5058527	2.6843898	3.28437795	NA	NA	NA	pheno	bin1	76-76-AL101-546_S22_L004
AL101_595	1996	470	M	7	3.352878	4	0.2833422798	1.097950431	2.184092526	3.093147742	NA	NA	NA	random	bin1	69-69-AL101-595_S15_L004
AL101_623	1996	220	M	6	2.235252	3	0.3882306	1.1764512	1.8940839	NA	NA	NA	NA	pheno	bin1	95-95-AL101-623_S41_L004

AL101_679	1996	510	F	7	3.4313135	4	0.2941162	1.2744875	2.0391795	2.8234765	NA	NA	NA	random	bin1	J32547-_L1_S14_L002
AL111_235	1997	270	M	5	2.3293683	2	0.7645091697	2.126296225	NA	NA	NA	NA	NA	pheno	bin1	81-81-AL111-235_S27_L004
AL111_28	1997	430	F	1	3.423462	4	0.5212224352	1.149051534	2.025651062	3.008856348	NA	NA	NA	pheno	bin1	67-67-AL111-28_S13_L004
AL114_826	1997	340	F	5	3.30582	3	0.8649636474	2.322368675	3.2	NA	NA	NA	NA	pheno	bin1	13-13-AL114-826_S13_L004
AL114_868	1997	350	F	4	3.246996	3	0.8176741991	2.287117683	3.128492023	NA	NA	NA	NA	pheno	bin1	5-05-AL114-868_S5_L004
AL114_937	1997	350	F	6	3.376407	3	0.8487897867	2.416796168	3.2	NA	NA	NA	NA	pheno	bin1	45-45-AL114-937_S45_L004
AL129_106	1998	400	F	1	2.9528847	4	0.399993	1.4823252	2.2234863	2.76465522	NA	NA	NA	pheno	bin1	18-18-AL129-106_S18_L004
AL129_148	1998	350	F	4	2.778504	5	0.4823439	1.0470411	1.623498	2.0726367	2.48439267	NA	NA	pheno	bin1	19-19-AL129-148_S19_L004
AL129_243	1998	240	M	6	2.1528984	2	0.4163059375	1.760383449	NA	NA	NA	NA	NA	pheno	bin1	42-42-AL129-243_S42_L004
AL129_266	1998	300	F	4	2.5293633	3	0.5343765927	1.033124708	1.971240748	NA	NA	NA	NA	pheno	bin1	50-50-AL129-266_S50_L004
AL129_339	1998	400	F	1	2.9881758	3	0.8267653782	1.759833805	2.326756389	NA	NA	NA	NA	pheno	bin1	15-15-AL129-339_S15_L004
AL129_369	1998	350	F	4	2.8705353	3	0.5551164224	1.665348665	2.23227125	NA	NA	NA	NA	random	bin1	23-23-AL129-369_S23_L004
AL129_377	1998	410	M	1	3.15288	5	0.611754	1.1646843	1.729377	2.388189	2.91759021	NA	NA	pheno	bin1	62-62-AL129-377_S8_L004
AL129_389	1998	410	F	1	2.8352403	2	0.4016601013	2.043735052	NA	NA	NA	NA	NA	pheno	bin1	3-03-AL129-389_S3_L004
AL129_401	1998	410	F	1	2.9764101	2	0.6640568246	2.430933466	NA	NA	NA	NA	NA	pheno	bin1	90-90-AL129-401_S36_L004
AL129_419	1998	270	M	6	2.4234843	3	0.5438058898	1.525022739	2.116114997	NA	NA	NA	NA	pheno	bin1	86-86-AL129-419_S32_L004
AL129_467	1998	270	M	6	2.2470147	3	0.5468895363	1.47423065	2.033013177	NA	NA	NA	NA	pheno	bin1	36-36-AL129-467_S36_L004
AL129_47	1998	400	F	1	3.30582	4	0.6493552721	1.157034585	2.160588787	2.739108187	NA	NA	NA	pheno	bin1	28-28-AL129-47_S28_L004
AL129_87	1998	350	F	4	2.8117119	2	0.8779157876	2.325297341	NA	NA	NA	NA	NA	pheno	bin1	34-34-AL129-87_S34_L004
AL130_199	1998	260	M	6	2.5528887	3	0.5964681468	1.049787613	2.290440306	NA	NA	NA	NA	pheno	bin1	98-98-AL130-199_S44_L004
AL130_206	1998	230	M	6	2.5293633	2	0.9928371908	2.115683232	NA	NA	NA	NA	NA	pheno	bin1	88-88-AL130-206_S34_L004
AL130_223	1998	420	F	1	3.176409	3	0.7851854841	2.010541617	2.938476416	NA	NA	NA	NA	pheno	bin1	8-08-AL130-223_S8_L004
AL130_244	1998	330	F	6	2.9881803	2	0.9249138092	2.027694449	NA	NA	NA	NA	NA	pheno	bin1	78-78-AL130-244_S24_L004

AL130_429	1998	410	F	1	3.129351	3	0.4978527531	1.967698629	2.939692628	NA	NA	NA	NA	pheno	bin1	48-48-AL130-429_S48_L004
AL130_440	1998	520	F	1	3.576402	3	0.6750192331	1.78820314	3.398767277	NA	NA	NA	NA	pheno	bin1	91-91-AL130-440_S37_L004
AL130_68	1998	420	F	1	3.23523	3	0.8549463871	2.113527618	2.680282951	NA	NA	NA	NA	pheno	bin1	46-46-AL130-68_S46_L004
AL133_100	1998	450	F	1	3.552873	3	0.8882189846	2.214623884	3.173899831	NA	NA	NA	NA	pheno	bin1	97-97-AL133-100_S43_L004
AL133_116	1998	340	F	6	2.9528862	3	0.6522451519	1.482374658	2.644552459	NA	NA	NA	NA	pheno	bin1	27-27-AL133-116_S27_L004
AL133_119	1998	320	F	6	2.6117169	3	0.6522451519	1.197758119	2.644552459	NA	NA	NA	NA	pheno	bin1	71-71-AL133-119_S17_L004
AL133_136	1998	470	F	1	3.399936	4	0.8854023731	1.605527096	2.679811202	3.175636356	NA	NA	NA	pheno	bin1	24-24-AL133-136_S24_L004
AL207_131	2002	390	M	7	2.9881758	5	0.6284651135	1.209496947	1.968402221	2.407140865	2.845879599	NA	NA	random	bin2	87-87-AL207-131_S33_L004
AL207_143	2002	410	F	7	2.9293536	4	0.3543567045	1.098505905	1.759972496	2.64036229	NA	NA	NA	random	bin2	107-107-AL207-143_S53_L004
AL207_146	2002	440	F	7	3.764634	4	0.694104	1.3882092	2.5528917	3.2	NA	NA	NA	random	bin2	101-101-AL207-146_S47_L004
AL207_149	2002	520	F	6	4.035219	6	0.5427495625	0.8731188615	1.392270316	2.312582686	3.138505331	3.763845804	NA	random	bin2	73-73-AL207-149_S19_L004
AL207_151	2002	640	F	6	4.117569	4	0.741164	2.164665	3.1411167	3.87051285	NA	NA	NA	random	bin2	55-55-AL207-151_S1_L004
AL207_197	2002	640	M	9	4.211685	4	0.599988	1.5	2.5764198	3.4822851	NA	NA	NA	random	bin2	57-57-AL207-197_S3_L004
AL207_20	2002	440	F	7	3.364644	4	0.5999913	1.6587927	2.270547	3	NA	NA	NA	random	bin2	51-51-AL207-20_S51_L004
AL207_231	2002	390	F	7	3.517578	4	0.7224642069	1.871302639	2.534550964	3.387294944	NA	NA	NA	random	bin2	106-106-AL207-231_S52_L004
AL207_249	2002	420	M	7	3.69405	4	0.647049	1.3646817	2.5999524	3.44699532	NA	NA	NA	random	bin2	33-33-AL207-249_S33_L004
AL207_25	2002	340	F	8	2.91759	3	0.858807	1.8117303	2.68230021	NA	NA	NA	NA	random	bin2	66-66-AL207-25_S12_L004
AL207_413	2002	430	F	7	3.894045	5	0.7338329108	1.810910549	2.603922628	3.065524183	3.633654307	NA	NA	random	bin2	72-72-AL207-413_S18_L004
AL207_462	2002	340	M	7	3.317583	4	0.6611543444	1.711921929	2.59739611	3.187715182	NA	NA	NA	random	bin2	40-40-AL207-462_S40_L004
AL207_473	2002	280	M	7	2.3764248	3	0.532034494	1.265062102	2.199079989	NA	NA	NA	NA	random	bin2	60-60-AL207-473_S6_L004
AL207_490	2002	340	M	6	2.9432226	3	0.8782172608	1.685232469	2.136207452	NA	NA	NA	NA	random	bin2	49-49-AL207-490_S49_L004
AL207_504	2002	300	M	7	2.7646581	4	0.5220840928	1.340798843	1.791689375	2.444288742	NA	NA	NA	random	bin2	16-16-AL207-504_S16_L004

AL207_516	2002	410	F	5	3.211704	4	0.7058661	1.788198	2.2823076	3.01170594	NA	NA	NA	random	bin2	108-108-AL207-516_S54_L004
AL207_539	2002	370	M	7	2.9999472	4	0.3188930824	1.027539658	1.99602551	2.740107833	NA	NA	NA	random	bin2	89-89-AL207-539_S35_L004
AL207_561	2002	320	M	7	3.69405	4	0.6941085	1.2588	2.4352512	3.50581758	NA	NA	NA	random	bin2	29-29-AL207-561_S29_L004
AL207_61	2002	360	F	7	3.376407	4	0.5194484002	1.35	2.184042542	3.104879284	NA	NA	NA	random	bin2	56-56-AL207-61_S2_L004
AL207_625	2002	410	M	7	3.752868	4	0.3433214951	2.048094775	3.042544295	3.634480872	NA	NA	NA	random	bin2	93-93-AL207-625_S39_L004
AL207_691	2002	270	M	6	2.36466	2	0.9340402522	1.927195885	NA	NA	NA	NA	NA	random	bin2	94-94-AL207-691_S40_L004
AL207_91	2002	300	M	8	2.5528917	4	0.5936973173	1.270509752	1.733591673	2.256044477	NA	NA	NA	random	bin2	30-30-AL207-91_S30_L004
AL318_200	2008	350	M	5	3.188178	4	0.3081526075	0.687416774	1.481498318	2.69039754	NA	NA	NA	random	bin3	58-58-AL318-200_S4_L004
AL318_234	2008	350	F	5	3.258759	6	0.5925007683	1.007250974	1.824903442	2.322606469	2.784755696	3.2	NA	random	bin3	63-63-AL318-234_S9_L004
AL318_29	2008	420	F	5	3.905808	6	0.270582	0.599988	1.8470214	2.541126	3.352875	3.9	NA	random	bin3	14-14-AL318-29_S14_L004
AL318_9	2008	240	M	5	2.2587816	3	0.4139116985	0.9697372029	1.785736611	NA	NA	NA	NA	random	bin3	105-105-AL318-9_S51_L004
AL320_1003	2008	480	F	5	3.517581	4	0.3316183982	1.136999585	2.250310503	3.3	NA	NA	NA	random	bin3	21-21-AL320-1003_S21_L004
AL320_1067	2008	310	M	5	2.847	3	0.8778179102	1.51839758	2.669068386	NA	NA	NA	NA	random	bin3	43-43-AL320-1067_S43_L004
AL320_1172	2008	310	M	7	2.8352403	3	0.7473292852	1.969170141	2.6	NA	NA	NA	NA	random	bin3	37-37-AL320-1172_S37_L004
AL320_132	2008	490	M	5	4.117566	5	0.3775425303	1.156218583	2.465818032	3.374278542	3.9	NA	NA	random	bin3	2-02-AL320-132_S2_L004
AL320_153	2008	310	M	5	2.5175976	3	0.6264462774	1.666575662	2.269380261	NA	NA	NA	NA	random	bin3	7-07-AL320-153_S7_L004
AL320_263	2008	300	M	5	2.847006	3	0.4863638243	1.589579793	2.6	NA	NA	NA	NA	random	bin3	1-01-AL320-263_S1_L004
AL320_331	2008	430	F	6	3.670518	6	0.6941019	1.4470317	2.0705454	2.5058325	3.1528773	3.5	NA	random	bin3	17-17-AL320-331_S17_L004
AL320_335	2008	490	M	5	4.505796	6	0.447054	1.247034	2.0940774	2.9175948	3.7999287	4.3	NA	random	bin3	31-31-AL320-335_S31_L004
AL320_379	2008	350	M	5	3.599931	3	0.75	2.45	3.4	NA	NA	NA	NA	random	bin3	6-06-AL320-379_S6_L004
AL320_398	2008	420	F	5	3.576399	6	0.6963977649	1.192131415	1.794099079	2.431479987	2.903610626	3.328529286	NA	random	bin3	44-44-AL320-398_S44_L004

AL320_487	2008	240	F	5	2.3881875	3	0.3294024	1.3293867	1.9646661	NA	NA	NA	NA	random	bin3	41-41-AL320-487_S41_L004
AL320_5	2008	370	F	5	3.235227	4	0.5058714	1.2235044	2.117601	3.03523188	NA	NA	NA	random	bin3	35-35-AL320-5_S35_L004
AL320_532	2008	390	F	5	3.094062	3	0.5646987	1.9881987	2.79995067	NA	NA	NA	NA	random	bin3	9-09-AL320-532_S9_L004
AL320_772	2008	430	F	5	3.505815	3	0.6514159446	2.226670653	3.375530361	NA	NA	NA	NA	random	bin3	10-10-AL320-772_S10_L004
AL320_865	2008	270	M	6	2.7293613	3	0.7541682573	1.831546336	2.5	NA	NA	NA	NA	random	bin3	12-12-AL320-865_S12_L004
AL320_879	2008	400	F	6	3.775266	4	0.494109	1.4940825	1.941141	3.1399809	NA	NA	NA	random	bin3	52-52-AL320-879_S52_L004
AL324_1047	2008	510	M	6	4.035219	5	0.6489411397	1.581052027	2.383374686	3.457073306	3.85	NA	NA	random	bin3	103-103-AL324-1047_S49_L004
AL324_1064	2008	440	M	6	3.870516	4	0.8048787446	1.941175432	2.923599148	3.468076549	NA	NA	NA	random	bin3	74-74-AL324-1064_S20_L004
AL324_1151	2008	480	F	8	4.105803	5	0.63528	1.8940794	2.5411272	3.2234691	3.8	NA	NA	random	bin3	32-32-AL324-1151_S32_L004
AL324_652	2008	220	M	6	2.2470147	2	0.7568868586	2.005097484	NA	NA	NA	NA	NA	random	bin3	80-80-AL324-652_S26_L004
AL435_298	2014	320	F	5	2.8940655	3	0.6286303753	2.348460953	2.8	NA	NA	NA	NA	random	bin4	J35428-S1-_L1_S125_L002
AL435_334	2014	310	M	5	2.8705371	3	0.8065983966	1.991122238	2.8	NA	NA	NA	NA	random	bin4	J35413-S1-_L1_S110_L002
AL435_403	2014	320	M	5	3.976395	5	0.7433609596	1.486723725	2.595865011	3.398224796	3.9	NA	NA	random	bin4	J35419-S1-_L1_S116_L002
AL435_413	2014	270	M	6	2.741127	4	0.7594484835	1.756220117	2.183408262	2.7	NA	NA	NA	random	bin4	J35426-S1-_L1_S123_L002
AL435_634	2014	390	F	5	3.023472	3	0.4742735811	2.07493539	3	NA	NA	NA	NA	random	bin4	J35414-S1-_L1_S111_L002
AL435_636	2014	410	M	5	3.494052	5	0.7764564	1.7529093	2.2470129	2.8234758	3.4	NA	NA	random	bin4	J35427-S1-_L1_S124_L002
AL435_721	2014	330	F	7	3.023472	4	0.8536846452	1.659944381	2.371347043	3	NA	NA	NA	random	bin4	J35424-S1-_L1_S121_L002

AL435_725	2014	310	M	6	2.5646601	2	0.7293984	2.36466204	NA	NA	NA	NA	NA	random	bin4	J35434-S1-_L1_S131_L002
AL435_730	2014	330	M	6	3.07053	3	0.6588114	1.141155	2.564661	NA	NA	NA	NA	random	bin4	J35423-S1-_L1_S120_L002
AL437_245	2014	320	M	6	2.8352403	3	0.7088129519	1.937410822	2.646223894	NA	NA	NA	NA	random	bin4	J35406-S1-_L1_S103_L002
AL437_251	2014	290	M	6	2.5646544	3	0.6470451	1.835256	2.39995323	NA	NA	NA	NA	random	bin4	J35395-S1-_L1_S92_L002
AL437_264	2014	320	M	6	3.294054	4	0.5213653754	1.421896367	2.606803913	3.1	NA	NA	NA	random	bin4	J35407-S1-_L1_S104_L002
AL437_288	2014	350	F	5	3.129351	3	0.6588099	2.2234857	2.92935297	NA	NA	NA	NA	random	bin4	J35396-S1-_L1_S93_L002
AL437_505	2014	230	M	6	2.1058419	3	0.6470508	1.2940959	1.92937512	NA	NA	NA	NA	random	bin4	J35398-S1-_L1_S95_L002
AL437_562	2014	430	F	6	3.563544	4	0.5210818746	1.338224334	2.79376555	3.4	NA	NA	NA	random	bin4	J35425-S1-_L1_S122_L002
AL437_573	2014	460	F	6	3.729339	5	0.793224895	1.988981835	2.770365723	3.1	3.5	NA	NA	random	bin4	J35399-S1-_L1_S96_L002
AL437_579	2014	420	F	5	2.9528877	3	0.566954701	1.996152263	2.8	NA	NA	NA	NA	random	bin4	J35397-S1-_L1_S94_L002
AL437_582	2014	320	F	5	3.223467	5	0.5214416408	1.113990801	1.730242567	2.405749341	3	NA	NA	random	bin4	J35432-S1-_L1_S129_L002
AL437_601	2014	340	F	6	3.446994	5	0.6278031271	1.445134023	2.262461899	2.736272713	3.4	NA	NA	random	bin4	J35405-S1-_L1_S102_L002
AL437_611	2014	310	F	5	3.282297	4	0.7764591	1.9764387	2.4940734	3.1	NA	NA	NA	random	bin4	J35433-S1-_L1_S130_L002
AL521_566	2019	300	M	5	2.3999532	3	0.7684568129	1.761544264	2.2	NA	NA	NA	NA	random	bin5	J35415-S1-_L1_S112_L002
AL521_726	2019	280	M	5	2.7764181	5	0.5339250681	0.8542825901	1.649239937	2.230626713	2.7	NA	NA	random	bin5	J35416-S1-_L1_S113_L002
AL521_847	2019	380	M	5	3.152883	5	0.5058723	1.0352757	1.72938	2.541129	2.92935663	NA	NA	random	bin5	J35420-S1-_L1_S117_L002
AL521_865	2019	270	M	5	2.6587704	3	0.6381021358	2.15065042	2.6	NA	NA	NA	NA	random	bin5	J35421-S1-_L1_S118_L002

AL522_473	2019	230	M	6	1.9785411	2	0.6056997595	1.836523718	NA	NA	NA	NA	NA	random	bin5	J35403-S1-_L1_S100_L002
AL522_510	2019	290	F	5	2.5646601	3	0.6263930347	1.938266975	2.3	NA	NA	NA	NA	random	bin5	J35418-S1-_L1_S115_L002
AL522_511	2019	250	M	6	2.741127	4	0.7148181803	1.199241196	2.27442566	2.7	NA	NA	NA	random	bin5	J35404-S1-_L1_S101_L002
AL522_519	2019	360	M	7	3.22347	5	0.7999857	1.8705519	2.4234837	2.811714	3.2	NA	NA	random	bin5	J35400-S1-_L1_S97_L002
AL522_531	2019	330	F	5	2.9293536	4	0.8386457811	1.535552474	2.374197954	2.85	NA	NA	NA	random	bin5	J35431-S1-_L1_S128_L002
AL522_532	2019	280	F	5	2.6705391	3	0.6617258696	2.06789564	2.469656008	NA	NA	NA	NA	random	bin5	J35411-S1-_L1_S108_L002
AL522_543	2019	220	M	6	2.1764298	2	0.5708696617	1.926680718	NA	NA	NA	NA	NA	random	bin5	J35401-S1-_L1_S98_L002
AL522_547	2019	370	F	7	2.8352403	4	0.6761882658	1.565906436	2.277681154	2.8	NA	NA	NA	random	bin5	J35402-S1-_L1_S99_L002
AL522_561	2019	300	F	5	2.43525	3	0.6029011425	1.66684797	2.246103743	NA	NA	NA	NA	random	bin5	J35408-S1-_L1_S105_L002
AL522_564	2019	300	M	6	3.011706	5	0.6639975875	1.470283612	2.098707352	2.572997001	2.9	NA	NA	random	bin5	J35409-S1-_L1_S106_L002
AL522_579	2019	320	F	5	2.8234746	3	0.7646895	2.0117226	2.62347654	NA	NA	NA	NA	random	bin5	J35412-S1-_L1_S109_L002
AL522_593	2019	330	F	7	2.6929806	3	0.5207789457	1.087076907	2.113984798	NA	NA	NA	NA	random	bin5	J35422-S1-_L1_S119_L002
AL522_594	2019	300	F	5	2.7646524	3	0.5576724108	1.95780449	2.645997957	NA	NA	NA	NA	random	bin5	J35429-S1-_L1_S126_L002
AL522_613	2019	270	M	5	2.3646681	3	0.8823399	1.9176096	2.18819544	NA	NA	NA	NA	random	bin5	J35410-S1-_L1_S107_L002
AL522_624	2019	300	F	5	2.8352343	5	0.4370949496	1.051398175	1.96103226	2.350880647	2.8	NA	NA	random	bin5	J35430-S1-_L1_S127_L002
AL522_682	2019	410	F	5	3.199944	4	0.4235259	0.9764517	2.4705456	3	NA	NA	NA	random	bin5	J35417-S1-_L1_S114_L002

Table S2. Estimated von Bertalanffy growth parameters for individual fish, catch years and all samples.

The Bayesian hierarchical model estimates parameters of nested levels at the same time. Here, three estimated von Bertalanffy parameters L∞, length at infinity, *k*, a growth coefficient, and t0, hypothetical length at age 0, were estimated for all samples, for each bin, based on the catch years, and for each individual. The individual parameters were used to calculate growth performance, Φ, which was used in genotype-phenotype association analysis.

	**L∞**	**k**	**t0**
All	7.4265107	0.1785009186	0.45453227
**group parameters**			
bin1	9.197300231	0.128748689	0.4674114129
bin2	8.07270249	0.1346183828	0.4368796201
bin3	8.355492139	0.1339595874	0.4697016901
bin4	5.601883356	0.2381987686	0.4521721634
bin5	4.794222888	0.2540789252	0.4417218798
**Individual parameters**			
AL097_334	9.450599562	0.1520235359	0.4475552977
AL097_430	9.087302412	0.1130445062	0.4992249003
AL097_552	8.991693021	0.1089703578	0.4471818953
AL099_189	8.87386943	0.1081162503	0.4034060656
AL099_326	9.001873984	0.1064093858	0.4727587402
AL099_328	9.294134062	0.1440551036	0.458454618
AL099_344	9.107911539	0.1144948398	0.5125570973
AL099_35	9.862030975	0.1545818366	0.6151723101
AL099_37	9.338815522	0.179830962	0.4050394258
AL099_577	9.886988158	0.1854323845	0.4736466419
AL099_591	8.922809483	0.08965639297	0.4861141298
AL099_597	9.519168066	0.1661813843	0.388586572
AL099_600	9.04811865	0.1077003178	0.5133851482
AL099_602	8.865169484	0.08686046859	0.4291770037
AL099_613	9.703720402	0.1723847808	0.4776966284
AL099_621	9.153721997	0.1159366327	0.5329026519
AL099_630	9.179612099	0.1220113227	0.510800712
AL099_636	9.269059273	0.1181639864	0.5105184667
AL099_78	9.017167028	0.09577655554	0.5042855393
AL101_105	9.300229768	0.155219942	0.4468083347
AL101_114	9.174572274	0.1124477676	0.4484334979
AL101_185	9.131668638	0.1103295181	0.5051449708
AL101_214	9.07790325	0.1186426579	0.4450988971
AL101_287	9.339071435	0.1272314452	0.5661345765

AL101_288	8.941416968	0.09725037072	0.4703341392
AL101_290	9.408169079	0.1379044579	0.5625363926
AL101_332	9.081519588	0.1115161007	0.5328694771
AL101_344	9.221909288	0.1375316857	0.377767302
AL101_422	9.036769493	0.1108161478	0.4243746867
AL101_423	9.507684883	0.154410608	0.4506158986
AL101_430	9.042936003	0.09719637536	0.4990055604
AL101_434	8.737914906	0.07886077014	0.4409890429
AL101_442	9.086019789	0.134018513	0.423991964
AL101_504	9.271457586	0.1230483967	0.5372382919
AL101_523	9.441536901	0.1366415583	0.5338619759
AL101_546	9.17519508	0.1277895548	0.4689240648
AL101_595	9.14239765	0.1131475528	0.5551555344
AL101_623	8.956316965	0.09746956772	0.4959048311
AL101_679	9.043583385	0.1060935329	0.5175851339
AL111_235	9.461991327	0.15358678	0.4465150743
AL111_28	9.07518508	0.1083889961	0.5008817759
AL114_826	9.526137235	0.1609316128	0.4221965564
AL114_868	9.475413075	0.1582214552	0.4294103049
AL114_937	9.532596612	0.1626247699	0.4209297711
AL129_106	9.002026372	0.1097054651	0.4810656196
AL129_148	8.794570505	0.07754371282	0.4536293287
AL129_243	9.245132909	0.1332643984	0.5029691637
AL129_266	8.950702232	0.09715883083	0.4819860694
AL129_339	9.109785129	0.1231829907	0.4162388817
AL129_369	9.09230404	0.1182275187	0.4620745171
AL129_377	8.851164347	0.08940231367	0.4486286454
AL129_389	9.416035586	0.1482700369	0.5093214217
AL129_401	9.662875585	0.1686834452	0.4689490516
AL129_419	9.040034327	0.1117776739	0.4653045182
AL129_467	9.010387282	0.1080124261	0.4631736665
AL129_47	9.002389964	0.1044357431	0.4646519995
AL129_87	9.591999296	0.1638570041	0.4284949264
AL130_199	9.058881759	0.1082911369	0.4867830913
AL130_206	9.47053881	0.1548214306	0.4071403752
AL130_223	9.374073623	0.1471718248	0.4393894005
AL130_244	9.408045541	0.1498888662	0.4189800309
AL130_429	9.406646004	0.1473586536	0.4953509983
AL130_440	9.583337471	0.1582945219	0.4978265952
AL130_68	9.249965364	0.1408448746	0.4081199343
AL133_100	9.49050287	0.1579455583	0.4210616173

AL133_116	9.200936866	0.1280703676	0.4736767546
AL133_119	9.163756832	0.1232225343	0.4880580957
AL133_136	9.117893336	0.1259574667	0.4129214144
AL207_131	7.649680902	0.1081416354	0.4202711597
AL207_143	7.795216507	0.1114914491	0.4674499961
AL207_146	8.161805783	0.138122074	0.4347289713
AL207_149	7.89575326	0.105939195	0.4810880663
AL207_151	8.598745009	0.1671428625	0.4109410267
AL207_197	8.306722064	0.1450613559	0.4501820786
AL207_20	8.02409794	0.1322939132	0.4251877645
AL207_231	8.245481996	0.1461482888	0.4120184535
AL207_249	8.29965245	0.1428372343	0.4499806396
AL207_25	8.297817071	0.1490380849	0.4018941439
AL207_413	8.011895365	0.1381896123	0.3946931393
AL207_462	8.177615271	0.1419191474	0.4198506593
AL207_473	7.936805647	0.1243798414	0.4476587427
AL207_490	7.984126882	0.1316682425	0.3958273588
AL207_504	7.721832776	0.1101414055	0.4334560669
AL207_516	8.026537311	0.1345181681	0.4081450666
AL207_539	7.871298514	0.1167095049	0.4777288743
AL207_561	8.267333165	0.1404821668	0.4525514602
AL207_61	8.045089124	0.1303392316	0.4531575304
AL207_625	8.516871434	0.1609191553	0.4573959705
AL207_691	8.375366546	0.1543463303	0.3911525867
AL207_91	7.652973621	0.1049047922	0.4250222648
AL318_200	8.004324218	0.1055665781	0.6565550876
AL318_234	7.713886643	0.09961048103	0.4345559428
AL318_29	8.296078913	0.1150727245	0.7374793351
AL318_9	7.904237062	0.104625754	0.5302913157
AL320_1003	8.518634158	0.133939944	0.6414515207
AL320_1067	8.470923633	0.1378787445	0.3915099997
AL320_1172	8.534894472	0.1434299212	0.3859437223
AL320_132	8.582672465	0.1386076274	0.6260217212
AL320_153	8.291394134	0.1303608222	0.4312805614
AL320_263	8.546185505	0.1403667817	0.5117499617
AL320_331	7.763856655	0.1113906249	0.3479169119
AL320_335	8.449163894	0.1279784037	0.5855312028
AL320_379	9.183784065	0.1743276474	0.4148729795
AL320_398	7.755807895	0.1029128544	0.3921064958
AL320_487	8.090126178	0.11787785	0.5356935788
AL320_5	8.270666667	0.125019782	0.527420293

AL320_532	8.739423257	0.1513249592	0.4638133059
AL320_772	9.15779552	0.1719636176	0.4647211654
AL320_865	8.446664257	0.1387328548	0.3884157674
AL320_879	8.265953535	0.126381176	0.505030336
AL324_1047	8.444693844	0.1370481296	0.4576930795
AL324_1064	8.589451741	0.1481852295	0.3686268299
AL324_1151	8.32059804	0.1364180702	0.3853341051
AL324_652	8.731817382	0.1512966595	0.4038428104
AL435_298	5.864809261	0.2652378212	0.4475483427
AL435_334	5.787893591	0.2559622744	0.4412232761
AL435_403	5.749631241	0.2394476233	0.4614281932
AL435_413	5.29856728	0.2187984678	0.4363076765
AL435_634	5.91046528	0.2627780346	0.467530867
AL435_636	5.352833388	0.2234446852	0.437419918
AL435_721	5.453864234	0.229488045	0.4387939227
AL435_725	6.01337452	0.2734623517	0.4443419378
AL435_730	5.47145833	0.2201121101	0.4649564816
AL437_245	5.699779538	0.2478242769	0.4472282347
AL437_251	5.547323367	0.2348889923	0.4491953529
AL437_264	5.561382498	0.229835008	0.469202689
AL437_288	5.9140313	0.2658132443	0.4494727246
AL437_505	5.214409517	0.1973634758	0.453997777
AL437_562	5.736571089	0.2388926366	0.478075704
AL437_573	5.475800125	0.2460417728	0.4290892014
AL437_579	5.789128394	0.254111154	0.4596674789
AL437_582	5.161057285	0.1825767766	0.4649460912
AL437_601	5.365783609	0.2146789286	0.4558613664
AL437_611	5.551650309	0.2405733583	0.436009765
AL521_566	4.785374419	0.2576090993	0.4200144405
AL521_726	4.599358919	0.187787258	0.4622705359
AL521_847	4.673541333	0.2088844679	0.4659267746
AL521_865	4.931469905	0.2952174229	0.4286036625
AL522_473	4.860981505	0.2732937974	0.4394298411
AL522_510	4.828798825	0.2692200531	0.4311532453
AL522_511	4.739056117	0.2372747965	0.4437710032
AL522_519	4.737025053	0.2642862543	0.4062924007
AL522_531	4.780649417	0.2570631968	0.4215520795
AL522_532	4.885994957	0.2850281813	0.4258320717
AL522_543	4.886445622	0.2783876422	0.4406260105
AL522_547	4.763554709	0.2521552246	0.436198989
AL522_561	4.783560822	0.2544689779	0.4399686355

AL522_564	4.647066793	0.229708281	0.4300718671
AL522_579	4.912403487	0.2922403256	0.4199102108
AL522_593	4.693241561	0.2201993833	0.4616249098
AL522_594	4.923511456	0.2888315373	0.4431665049
AL522_613	4.792925566	0.2659231351	0.4057731667
AL522_624	4.62913837	0.2064427007	0.4635240498
AL522_682	4.845561151	0.2483105756	0.4853192219

Table S3. A list of genes intersecting or neighbouring with GWA outlier SNPs with its position and description.

A list of genes unique in the "ensembl_gene_id" located at or 10 Kb surrounding regions of GWA outlier SNPs was subjected to a search for their functional annotations in the literature. Columns, ensembl_gene_id, chromosome_name, external_gene_name, description, start_position, and end_position, are annotations in the gadMor3.0 reference genome extracted from Ensembl database. When the external_gene_name were not provided by the database, search in the NCBI gene database or orthologs' name were filled in (Genes, NCBI). Functional annotations of the genes, in the most relevant context to this study, "known biological functions from literature", were listed by searching the gene names with or without keywords (e.g., fish, growth, weight, maturity, and reproduction) in the literature search. When there were no search results which showed direct or indirect biological relevance in the targeted context, they were marked as "Not found in literature". Some genes were catalogued with only weak matches to orthologs in other species in the database, thus marked as "NA (Not applicable)". Rows containing genes which are most relevant to this study are highlighted in grey.

ensembl_gene_id	external_gene_name	chromosome_name	known biological functions from literature	description	start_position	end_position
At the outlier loci						
ENSGMOG00000032747	HEPACAM	3	cell-adhesion, cell motility, cancer suppressor gene	carcinoembryonic antigen-related cell adhesion molecule 6-like [Source:NCBI gene (formerly Entrezgene);Acc:115540996]	13790699	13856484
ENSGMOG00000015953	mnd1	3	meiotic arrest, recombination	meiotic nuclear divisions 1 homolog (*S. cerevisiae*) [Source:NCBI gene (formerly Entrezgene);Acc:115540214]	20677607	20683019
ENSGMOG00000012895	NA	3	NA	NA	21756782	21778636
ENSGMOG00000016255	haspin	3	mitosis, ciritical larval growth and survival in zebrafish	histone H3 associated protein kinase [Source:NCBI gene (formerly Entrezgene);Acc:115540151]	25267491	25278374
ENSGMOG00000016202	ncapg	3	DEGs in salmon puberty	non-SMC condensin I complex, subunit G [Source:NCBI gene (formerly Entrezgene);Acc:115540152]	25305001	25318049
ENSGMOG00000007843	PDE4D	6	Transcriptomic response in a rainbow trout line selectively bred for fast growth (growth line, GL). Several key components of the cAMP signaling pathway were reduced in the GL, including adenylate cyclase-type 6 (adcy6) and phosphodiesterase 4D (pde4d)	phosphodiesterase 4D, cAMP-specific [Source:NCBI gene (formerly Entrezgene);Acc:115545011]	16947270	17078303
ENSGMOG00000013441	trpm1b	9	retina pigment development	transient receptor potential cation channel subfamily M member 1-like [Source:NCBI gene (formerly Entrezgene);Acc:115550405]	21730570	21750419
ENSGMOG00000037198	ciart	9	Transcriptional changes in the process of reproductive hormone affecting circadian rhythm in zebrafish. Generally involved in circadian regulation of gene expression	circadian-associated transcriptional repressor-like [Source:NCBI gene (formerly Entrezgene);Acc:115550867]	21823470	21831852
ENSGMOG00000017135	coro1ca	13	Not found in literature	coronin, actin binding protein, 1Ca [Source:NCBI gene (formerly Entrezgene);Acc:115556521]	25841273	25924738
ENSGMOG00000008588	ercc5	14	higher rate of malformations and decreased embryo viability in fish, COFS syndrome in human.	excision repair cross-complementation group 5 [Source:NCBI gene (formerly Entrezgene);Acc:115558732]	21543979	21552546

ENSGMOG00000016695	PPFIBP2 (orthologs)	14	human fetal abnormality	PPFIA binding protein 2a [Source:ZFIN;Acc:ZDB-GENE-070705-277]	22269074	22285193
ENSGMOG00000018629	kcnk2b (ortholog)	21	increased expression during puberty in zebrafish	NA	4143319	4168663
ENSGMOG00000013114	snx17	21	intracelluar protein transport, in a conserved genomic region (together with atraid) associated with miR-133b which is involved in oogenesis in a tilapia	sorting nexin 17 [Source:NCBI gene (formerly Entrezgene);Acc:115534617]	4307230	4333109
5 Kb up- and down-stream						
ENSGMOG00000026566	NA	3	NA	NA	13810816	13811780
ENSGMOG00000015878	TMEM131L	3	Not found in literature	transmembrane 131 like [Source:NCBI gene (formerly Entrezgene);Acc:115540213]	20642815	20676221
ENSGMOG00000030622	NA	3	NA	NA	25275974	25276992
ENSGMOG00000036363	fam184b	3	chicken body weight at first egg	family with sequence similarity 184 member B [Source:NCBI gene (formerly Entrezgene);Acc:115540150]	25280244	25303554
ENSGMOG00000022331	lcorl	3	human: Expression of the LCORL gene was significantly associated with length of the neonate at birth	ligand dependent nuclear receptor corepressor-like [Source:NCBI gene (formerly Entrezgene);Acc:115540149]	25319776	25337592
ENSGMOG00000036563	NA	13	NA	NA	25928210	25928944
ENSGMOG00000033950	cenpq	14	Not found in literature	centromere protein Q [Source:NCBI gene (formerly Entrezgene);Acc:115559135]	21398766	21402387
ENSGMOG00000025157	gmo-mir-155	14	Not found in literature	gmo-mir-155 [Source:miRBase;Acc:MI0036008]	21410103	21410161
ENSGMOG00000003226	ephrin-B2a-like	14	angiogenesis	ephrin-B2a-like [Source:NCBI gene (formerly Entrezgene);Acc:115559324]	21411256	21419004
ENSGMOG00000025102	trmt10c	14	Not found in literature	tRNA methyltransferase 10C, mitochondrial RNase P subunit [Source:ZFIN;Acc:ZDB-GENE-041114-12]	21536364	21543958
ENSGMOG00000035263	blzf1	14	heart function, development in medaka	basic leucine zipper nuclear factor 1 [Source:NCBI gene (formerly Entrezgene);Acc:115558734]	21538764	21543958
ENSGMOG00000030760	mettl21e	14	linked to growth in pupfishes, intramuscular fat deposition in cattle	methyltransferase like 21e [Source:NCBI gene (formerly Entrezgene);Acc:115558735]	21552352	21557877
ENSGMOG00000024865	NA	14	differentially expressed in response to temperature in stickleback, also generally skeletal muscle development related	protein-lysine methyltransferase METTL21C-like [Source:NCBI gene (formerly Entrezgene);Acc:115558577]	21560583	21565349
ENSGMOG00000013100	atraid	21	bone differentiation	all-trans retinoic acid-induced differentiation factor [Source:NCBI gene (formerly Entrezgene);Acc:115534618]	4334479	4337000

Table S4. A list of enriched GO terms for outlier windows F_{st} between 1996 and 2019.

F_{st} values in non-overlapping 50Kb windows along the genome were calculated for 1996 and 2019 to identify regions that are the most differentiated over time. The windows with highest 5% F_{st} were assigned as outlier windows. The genes residing in these outlier windows were subjected to gene ontology (GO) enrichment test to identify any biological functions that have differentiated over time. P values are adjusted using false discovery rate. Only biological processes were presented among GO categories for the analysis.

GO.term	GO.name	p.value.adjusted
GO:0006468	protein phosphorylation	0.028
GO:0006541	glutamine metabolic process	0.028
GO:0019217	regulation of fatty acid metabolic process	0.028
GO:0016079	synaptic vesicle exocytosis	0.028
GO:0031998	regulation of fatty acid beta-oxidation	0.028
GO:1990504	dense core granule exocytosis	0.028
GO:0006171	cAMP biosynthetic process	0.028
GO:0034220	ion transmembrane transport	0.029
GO:0019395	fatty acid oxidation	0.042
GO:0042157	lipoprotein metabolic process	0.042
GO:0051301	cell division	0.042
GO:0061074	regulation of neural retina development	0.042
GO:0033077	T cell differentiation in thymus	0.042
GO:0006044	N-acetylglucosamine metabolic process	0.042
GO:0006432	phenylalanyl-tRNA aminoacylation	0.042
GO:0070509	calcium ion import	0.042
GO:0062012	regulation of small molecule metabolic process	0.042
GO:0007166	cell surface receptor signaling pathway	0.042
GO:0030178	negative regulation of Wnt signaling pathway	0.042
GO:0006814	sodium ion transport	0.042
GO:0005975	carbohydrate metabolic process	0.042
GO:0007264	small GTPase mediated signal transduction	0.042
GO:0140013	meiotic nuclear division	0.042
GO:0007169	transmembrane receptor protein tyrosine kinase signaling pathway	0.042
GO:0009190	cyclic nucleotide biosynthetic process	0.042
GO:0060027	convergent extension involved in gastrulation	0.042
GO:0006796	phosphate-containing compound metabolic process	0.042
GO:0030968	endoplasmic reticulum unfolded protein response	0.042
GO:0030098	lymphocyte differentiation	0.042
GO:0061982	meiosis I cell cycle process	0.042
GO:0001702	gastrulation with mouth forming second	0.042
GO:0050794	regulation of cellular process	0.042

GO:0010033	response to organic substance	0.042
GO:0009081	branched-chain amino acid metabolic process	0.042
GO:0007099	centriole replication	0.042
GO:0035567	non-canonical Wnt signaling pathway	0.042
GO:0032007	negative regulation of TOR signaling	0.042
GO:0021952	central nervous system projection neuron axonogenesis	0.042
GO:0055074	calcium ion homeostasis	0.042
GO:0009062	fatty acid catabolic process	0.042
GO:0050769	positive regulation of neurogenesis	0.042
GO:0042110	T cell activation	0.042
GO:0035967	cellular response to topologically incorrect protein	0.042
GO:0030433	ubiquitin-dependent ERAD pathway	0.042
GO:0060028	convergent extension involved in axis elongation	0.042
GO:0016579	protein deubiquitination	0.043
GO:0030001	metal ion transport	0.043
GO:0007154	cell communication	0.043
GO:0048729	tissue morphogenesis	0.045
GO:0048585	negative regulation of response to stimulus	0.045
GO:0009247	glycolipid biosynthetic process	0.047
GO:0023052	signaling	0.047
GO:0065004	protein-DNA complex assembly	0.048
GO:0090162	establishment of epithelial cell polarity	0.048
GO:0035825	homologous recombination	0.048
GO:0007131	reciprocal meiotic recombination	0.048
GO:0006040	amino sugar metabolic process	0.048
GO:0006801	superoxide metabolic process	0.048
GO:0098655	cation transmembrane transport	0.048
GO:0048285	organelle fission	0.048
GO:0023057	negative regulation of signaling	0.048
GO:0010648	negative regulation of cell communication	0.048
GO:0051716	cellular response to stimulus	0.048
GO:0023061	signal release	0.048
GO:0032402	melanosome transport	0.048
GO:0006284	base-excision repair	0.048
GO:0036211	protein modification process	0.048
GO:0043632	modification-dependent macromolecule catabolic process	0.048
GO:0006511	ubiquitin-dependent protein catabolic process	0.048
